# Genome Editing Approaches with CRISPR/Cas9 for Cancer Treatment: Critical Appraisal of Preclinical and Clinical Utility, Challenges, and Future Research

**DOI:** 10.3390/cells11182781

**Published:** 2022-09-06

**Authors:** Sergiu Chira, Andreea Nutu, Ecaterina Isacescu, Cecilia Bica, Laura Pop, Cristina Ciocan, Ioana Berindan-Neagoe

**Affiliations:** Research Center for Functional Genomics, Biomedicine and Translational Medicine, “Iuliu Hatieganu” University of Medicine and Pharmacy, 400337 Cluj-Napoca, Romania

**Keywords:** malignant disorders, CRISPR/Cas9, genome editing, cancer targets, clinical trials

## Abstract

The increasing burden on human malignant diseases became a major concern for healthcare practitioners, that must deal with tumor relapse and the inability to efficiently treat metastasis, in addition to side effects. Throughout the decades, many therapeutic strategies have been employed to improve the clinical outcomes of cancer patients and great efforts have been made to develop more efficient and targeted medicines. The malignant cell is characterized by genetic and epigenetic modifications, therefore targeting those specific drivers of carcinogenesis is highly desirable. Among the genome editing technologies, CRISPR/Cas9 stood as a promising candidate for cancer treatment alternatives, due to its low complexity design. First described as a defense mechanism of bacteria against invading foreign DNA, later it was shown that CRISPR components can be engineered to target specific DNA sequences in a test tube, a discovery that was awarded later with the Nobel Prize in chemistry for its rapid expansion as a reliable genome editing tool in many fields of research, including medicine. The present paper aims of describing CRISPR/Cas9 potential targets for malignant disorders, and the approaches used for achieving this goal. Aside from preclinical studies, we also present the clinical trials that use CRISPR-based technology for therapeutic purposes of cancer. Finally, a summary of the presented studies adds a more focused view of the therapeutic value CRISPR/Cas9 holds and the associated shortcomings.

## 1. Introduction

Comparing medicine of today with the one that prevailed over the past century, we have witnessed a rapid expansion of new treatment methods and strategies, spanning from basic surgery to advanced drug formulations, meant to ease the increasing burden of human diseases in both developed and underdeveloped countries. Among the diverse array of chronic or acute disorders, cancer is ranked as the first cause of mortality in developed countries, aside from the elevated costs for diagnosis and management of the disease [1]. This malignant disease requires complex and multi-disciplinary approaches for treatment, spanning from surgical resection to radiotherapy and chemotherapeutic agents, either as monotherapy or in combination. Despite recent advances, the health care practitioner must deal with many limitations in daily practice, such as low tumor specificity of drugs and hence side effects, tumor relapse, and inability to treat metastases, in addition to patient’s poor quality of life as a result of treatment regimens [2,3]. In addition, the aforementioned therapies pose also a risk for secondary malignancies in the long term [4,5,6]. Bearing these in mind, innovative treatment approaches are required to accomplish complete and sustained remission and avoid the risk of secondary malignancy occurrence. Having the experience of more than two decades of research and clinical trials, cell and gene therapy are becoming new and promising players on the table for cancer treatments [7,8]. Generally described as uncontrolled proliferation of cells, genetic and epigenetic alterations stand as the root for carcinogenesis. Such malignant transformation of the cells is driven by impairment of function in genes that are involved in the cell cycle regulation and homeostasis, and activation of proto-oncogenes [9]. From a classical point of view, gene therapy implies the transfer of a therapeutic gene to compensate for the defective gene or genes, or to deliver a gene whose function would interfere with the expression of tumor-promoting genes. However, this leads to a partial effect, as the core “issue” persists within the cell, sustaining its malignant behavior. In addition, the transferred genetic material can have a genotoxic effect on the genome, with the potential of activating proto-oncogenes or alter tumor suppressor genes expression by insertional mutagenesis [10]. Therefore, more precise targeting of the desired genomic loci is needed for achieving a better and safe outcome. CRISPR/Cas9 initially described as a defense mechanism of bacteria against bacteriophage infection [11], emerged as a promising tool for genome editing due to the ease of adapting it to mammalian cells, versatility, and flexibility for targeting virtually any genomic loci [12]. Biomedical research for the treatment of human diseases is one of the fields that took full advantage of what CRISPR/Cas9 can offer, that is molecular scissors to cut into the genetic material of diseased cells and repair the “mistakes” that characterize and promote the afflicted status of the cells [13,14].

In the present paper, we focus our view on CRISPR/Cas9 technology as a potential treatment option for malignant disorders, by describing the approaches by which this goal can be achieved, and nonetheless the limitations that can arise on the pathway toward clinical practice. Moreover, we examine from a critical point of view how the balance between bench and bedside can be shifted from laboratory knowledge to a CRISPR/Cas9 medical device.

## 2. From Bacteria Defense System to Mammalian Genome Engineering

The clustered regularly interspaced palindromic repeats or simply CRISPR are genetic structures first discovered in 1987 in the *Escherichia coli* genome [15] and later in other bacteria and archaea genomes as well [16,17]. Their function remained unknown until 2005, when a glimpse came from the observation that the spacer sequences between the repeats were homologous to sequences found in the genome of bacteriophages, archaeal viruses, and plasmids. If such matching sequences exist, the invading exogenous genetic material is not able to infect bacterial cells, suggesting their role as a prokaryotic defense mechanism. Adjacent to the CRISPR locus, CRISPR-associated (Cas) genes were also discovered and characterized as encoding sequences for nucleases that recognize a short stretch of 2–5 nucleotides, named protospacer adjacent motif (PAM) in the invading genome, which carry sequences with homology in the spacer sequences of CRISPR array locus [17,18,19]. This array locus encodes two types of RNA sequences, a CRISPR RNA (crRNA) that contains the variable spacer sequences and a trans-activating crRNA (tracrRNA), which together with the Cas nuclease constitutes the effector ribonucleoprotein (RNP) complex. Upon PAM binding, cleavage of the invading phage genome occurs, resulting in a double-strand break (DSB), thus interfering with the expression and multiplication of the phage. The cleavage is mediated by two domains of the Cas nuclease, the HNH domain which cleaves the target strand, and the RuvC domain which is responsible for the cleavage of the opposite strand. Class II CRISPR systems gained particular interest due to their low level of complexity [20]. In 2012, the crRNA and tracrRNA were fused into a chimeric single guide RNA (sgRNA or just gRNA) thus simplifying the system even further [12]. This study showed that Cas9 and the new chimeric crRNA/tracrRNA can cut virtually any DNA molecule in a test tube at a predetermined site, an observation that was granted in 2020 with the Nobel prize in chemistry to Emmanuel Charpentier and Jennifer A. Doudna [21]. By optimizing Cas9 for human codons and adding nuclear localization signals to it, CRISPR/Cas9 was first used in human and mouse cells in 2013 [22,23]. Since then, CRISPR/Cas9 has been readily accepted as a new versatile tool for gene editing, as it can be easily re-programmed by only modifying the crRNA sequence while leaving the remaining components, tracrRNA, and Cas nuclease, unchanged. As opposed to other genome engineering tools, namely the zinc finger nucleases (ZFNs) [24] and transcription activator-like effector nucleases (TALENs) meganucleases [25], CRISPR/Cas9 avoids the more intricate field of protein engineering which makes it cost-effective and ready to use. In mammalian cells, including human cells, upon Cas9 PAM binding and cleavage, the DSBs are repaired by non-homologous end joining (NHEJ), by which short insertions or deletions (indels) are generated in the targeted locus. This event can lead to a reading frame shift and impaired expression of the edited genomic locus [12,22].

## 3. Approaches for Therapeutic Genome Editing in Human Malignant Cells Using CRISPR/Cas9

### 3.1. CRISPR/Cas9 Knockout of Oncogenes—Double-Strand Break (DBS) Approach

**Figure 1:**
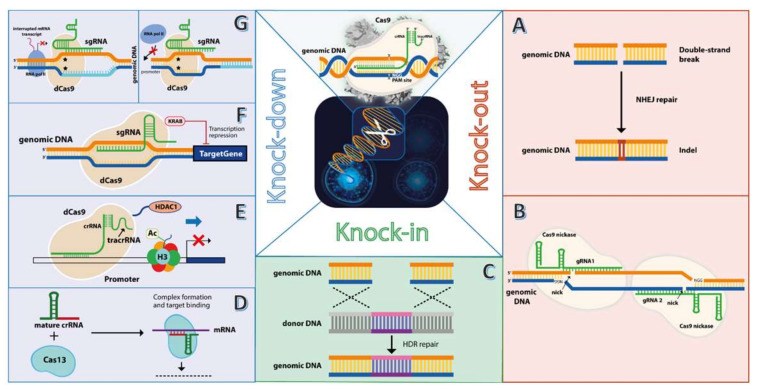
Approaches for gene editing in tumor cells using CRISPR/Cas system. The classical CRISPR/Cas9 effector ribonucleoprotein (RNP) (upper middle image) features the Cas9 nuclease and guide RNA (gRNA), comprising two sequences, the CRISPR RNA (crRNA) which shares homology with the target genomic locus, and the transactivating crRNA (tracrRNA). Upon recognition and binding to the protospacer adjacent motif (PAM), cleavage of both genomic DNA strands occurs 3 nucleotides upstream of PAM, resulting in a double-stand break (DSB). This breach in the genomic DNA is repaired by non-homologous end joining (NHEJ), which leads to small insertions or deletions (indels) (**A**). This process is the most explored feature of CRISPR/Cas9 to knockout oncogenes, as indels can lead to open reading frame shifts, with deleterious effects on oncogene expression. Because Cas9 nuclease exhibits unspecific cleavage activity (off-target) effects, a nickase-engineered variant of Cas9 has been developed, which mediates cleavage of the opposite genomic DNA strand that harbors the PAM sequence (**B**). By using two gRNAs with different sequence specificity (gRNA1 and gRNA2), an upstream and downstream cleavage of both gDNA strands is mediated by the Cas9 nickase, an enhanced “on-target” oncogene knockout is achieved, while off-target effects are minimized. Besides the NHEJ, the genomic DNA breach mediated by Cas9 nuclease can be filled by exogenous sequence by homology-directed repair (HDR) between the genomic DNA and donor DNA (**C**). HDR-mediated knock-in mostly includes suicide genes, such as thymidine kinase, inserted into the tumor genome for achieving a therapeutic. Besides knocking out oncogenes in the tumor genes, another approach uses a different type of CRISPR system, named Cas13, that targets the oncogene transcript, resulting in a knockdown effect (**D**). Fusion peptides to a dead Cas9 (dCas9), such as the histone deacetylase (**E**) that removes acetyl restudies from histone proteins (i.e., H3), or transcriptional repressor Kruppel-associated box (KRAB) (**F**) can result in a stronger repression effect compared to dCas9 alone, which interferes with oncogene transcription by stearic repulsion of RNA polymerase II (RNA pol II) (**G**, right panel) or blocking the transcription site (**G**, left panel).

The ability of Cas9 to generate DSBs in the target locus, which is further repaired by NHEJ (Figure 1A), is a process with deleterious consequences for the encoding DNA sequence, as indels may lead, as mentioned above, to frame shifts of the open reading frame (ORF) and impairment of gene function. Moreover, this propriety of CRISPR/Cas9 is the most widely used approach for achieving a therapeutic effect by knocking-out genes that promote and sustain tumor cell growth, proliferation, and invasion (Supplementary Table S1).

Perhaps one of the most representative oncogenes is KRAS, belonging to the RAS family of small GTPases, which were originally described as a viral oncogene of RNA tumorigenic viruses. Now is well established to be a highly mutated proto-oncogene in the deadliest types of human malignancies, such as colon cancer, lung cancer, and pancreatic cancer [26]. Being a difficult druggable target [27], KRAS represents an appealing objective for gene editing by CRISPR/Cas9. The knocking-out capabilities of *Streptococcus pyogenes* Cas9 (SpCas9) enabled researchers to induce inhibition of proliferation and increased apoptosis in colorectal cell line SW-480 in vitro using SpCas9/gRNA polymeric nanoparticles. In addition, systemic administration of these nanoparticles was efficient in reducing the tumor growth and metastatic potential of SW-480 xenografted mice [28]. Similar results have been obtained on a lung cancer cell line, A549, which expresses the mutant variant G12S, a feature that was exploited to design a mutation-specific gRNA, as the G12S mutation is localized next to a PAM motif. Transduction of the A549 cells in vitro with an adenoviral vector expressing SpCas9 and G12S-specific gRNA showed significant inhibition of cell proliferation and clonogenic capacity, while in vivo, intratumor injection with the same vector resulted in a reduced xenograft tumor growth [29]. Mutation-specific directed CRISPR/Cas9 systems have been also developed to target the epithelial growth factor receptor (EGFR) harboring the L858R substitution, which accounts for approximately 42% of lung adenocarcinoma in Asians. This mutation leads to a PAM motif in the EGFR sequence, that can be used to design a gRNA with specificity for the L587R substitution. By transduction of the L858-positive lung cancer cell line H1975 with lentiviral [30] or adenoviral [31] vectors expressing SpCas9/L587R-gRNA, a decrease in cell proliferation and tumor burden was obtained in vitro, and in vivo xenograft animal models, respectively.

The tumor suppressor gene *TP53*, which is also called the “guardian of the genome”, is a key player in cell homeostasis, being involved in DNA damage response and apoptosis; and it is no surprise that almost half of the human malignant tumors harbor a mutated form of *TP53*, that reflects in the malignant phenotype of cancer cells, and often it is associated with a very poor prognosis [32]. Mutations in the coding sequence of *TP53* can translate to a lack of function for p53 protein, however mutated variants of *TP53* can also lead to a gain of function, by which p53 acquires pro-carcinogenic proprieties [33]. In this instance, knocking out the mutated *TP53* by CRISPR/Cas9 can have a therapeutic value, as studies show in osteosarcoma in vitro models [34]. Two human osteosarcoma cell lines, KHOS and KHOSR2 (a paclitaxel and doxorubicin-resistant variant of parental KHOS cell line), both harbor a mutated form of *TP53*, where transfected with a Cas9/gRNA-expressing plasmid vector.

Targeting the fifth exon of *TP53* was found to inhibit proliferation, cell clone formation capability, and migration. Moreover, gene expression analysis pointed out that *TP53* knockout is associated with reduced expression levels of other oncogenes, such as the anti-apoptotic genes *Bcl-2*, *Survivin,* and *IGF-1R*. Furthermore, abrogating mutated *TP53* expression in KHOSR2, renders this cell line sensitive to doxorubicin treatment [34]. Interestingly, some malignant cells can compensate for p53 function impairment, by upregulating Ral-interacting protein (RLIP), which is involved in cell plasticity, migration, and endocytosis. By knocking out RLIP expression with CRISPR/Cas9, researchers were able to inhibit cell proliferation, and cell survival in *TP53* mutant breast cancer cell line MDA-MB-231 in vitro and circumvent xenograft tumor formation in mice models [35]. Not always a loss of function or an oncogenic gain of function of p53 protein is a result of mutations arising in the *TP53* locus, but repression of p53 by another factor results in a malignant phenotype of the cell. The *E6* gene harbored by the human papillomavirus (HPV) is well known to have such an inhibition effect on the p53 pathway in cervical cancers, by promoting degradation of p53 protein and thus cell cycle progression and proliferation [36]. Targeting the *E6* gene by CRISPR/Cas9 would be a rational approach for induce cell cycle arrest in HPV-positive cervical cancer cells. In vitro knocking out of the *E6* gene in models, such as HeLa, HCS-2, SKG-I, or Caski cell lines, leads to activation of the p53 signaling pathway, and subsequently reduced cell proliferation and induction of apoptosis [37,38,39]. Moreover, as a proof of concept, *E6* knockout in an SKG-I tumor xenografted mouse model that stably expresses Cas9, by intratumor injection of an adeno-associated viral (AAV) vector harboring the E6-gRNA, resulted in a reduction in the tumor mass compared to non-targeted control [37]. In other studies, the HPV16 E7 protein, which induces the expression of cell cycle-related genes by targeting the retinoblastoma (RB) pathway, was also targeted for knockout by CRISPR/Cas9. This approach proved to be highly efficient for eradicating established Caski tumor xenografts in Rag1 mice, with 4 out of 5 mice showing no tumors present at day 77 evaluation point. The tumor inhibition effect was also confirmed on the more aggressive HPV18-E7 HeLa tumor xenografts in Rag1 mice, where tumor volumes were monitored for 46 days [40]. Besides inhibition of the p53 and pRB pathways, a direct connection between HPV16-E6/E7 and activation of the programmed death (PD-1/PD-L1) pathway was also observed, as E6/E7 knockout by CRISPR/Cas9 was associated with decreased expression of PD-L1 in SiHa cells that stably express E6/E7 proteins [41]. These immune checkpoint molecules (PD-1 and PD-L1) are well characterized for their role in promoting an immune suppressive tumor microenvironment, limiting the efficiency of treatments [42]. To test the hypothesis that simultaneous knockout of HPV E6/E7 oncogenes and PD-1 can result in enhanced inhibition of tumor growth, a humanized immune-competent model was generated by intraperitoneal injection of human peripheral blood mononuclear cells into SCID mice. Cervical cancer orthotopic tumors were established in these mice models by implanting tumor fragments in the uterus, which originate from SiHa tumor xenografts of SCID mice. In situ electroporation of orthotopic tumors with Cas9-expressing plasmid and E6-, E7-, and PD-1-targeting gRNA plasmids, showed a marked tumor inhibition growth and increased survival when E6/E7 and PD-1 were simultaneously knocked out. In addition, for this treatment group, increased infiltration of CD4+ and CD8+ T cells was also observed in the orthotopic tumors, suggesting the importance of combined E6/E7 and PD-1 inhibition as an efficient approach in cervical cancer treatment [41]. As E6 and E7 upregulate the PD-1/PD-L1 pathway, the expression of these HPV oncogenes is regulated by the endogenous mixed lineage leukemia 5 (MML5) factor as a previous study shows [43]. Bearing this in mind, Pirouzfar M., and colleagues [44] used the knocking out potential of the CRISPR/Cas9 system to obtain an impairment of function in *HPV18-E6* and *MLL5* genes in HeLa cells. By this means, not only a reduction in cell viability was observed, but in addition, the double knockout enhanced the pro-apoptotic effect of cisplatin in HeLa cells in vitro. However, the authors did not test such synergistic effects on in vivo animal models. Even so, these studies highlight the importance of knocking out multiple oncogenes for achieving an enhanced anti-tumor outcome. In addition, a greater challenge would be targeting the oncogenic viruses before the malignant transformation of the infected cell, and this subject has been a matter of debate in recent reviews [45,46,47]. The presented strategies focus on disrupting the virus life cycle, which translates into a prophylactic treatment, an approach that raises important safety and ethical issues for the “immunization” of high-risk susceptible individuals; and for a curative purpose, this would mean identification of the infected individuals, such as regular gynecological screens for HPV, and immediate intervention by CRISPR/Cas9 for targeting the virus before exerting its oncogenic potential. Therefore, the time frame between virus infection and malignant transformation must be carefully evaluated to achieve an effective CRISPR/Cas9 intervention. It is worthwhile to mention a study that shows Kaposi’s Sarcoma-associated Herpesvirus (KSHV)-transformed precursor mesenchymal cells can be reversed to a “normal” state by targeting the major latent viral gene *LANA* [48], suggesting that the used approach might pave the pathway for new therapies of malignant diseases of viral etiology.

Impairment of the PD-1/PD-L1 immune checkpoint axis has been a matter of debate for researchers that aim of improving existing immunotherapies for both solid and hematological malignant disorders. Despite significant progress in the field of chimeric antigen receptor (CAR) T cell therapy for myeloproliferative cancers [49,50], for solid tumors, this treatment approach is hampered by the immune suppressive tumor microenvironment [51]. Moreover, the PD-1/PD-L1 immune checkpoint axis is one of the major factors that limit the therapeutic efficiency of CAR-T cells. This observation has been supported by studies that used CRISPR/Cas9 to knockout the PD-1 receptor in Glypican-3 (GPC3) targeted CAR-T cells in both in vitro and in vivo models of human hepatocellular carcinoma. Disruption of PD-1 showed to enhance the cytokine release of anti-GPC3 CAR-T cells and promote PLC/PRF/5 tumor cells lysis. Furthermore, the PD-1 edited CAR-T cells treatment of NSG mice harboring PLC/PRF/5 tumor led to inhibition of tumor growth due to enrichment of modified CAR-T cells in the tumor stroma and enhanced persistence compared to non-edited CAR-T cells. This effect of PD-1 knockout prolonged the survival rates of the PLC/PRF/5 tumor-bearing mice [52]. The anti-tumor efficiency of disrupting PD-1 receptors in EGFRvIII targeted CAR-T cells has also been tested on the DK-MG in vitro human glioblastoma model, displaying enhanced inhibitory effects on cell proliferation, when compared to unedited CAR-T cells [53]. With such a work frame in place, researchers have shown that an impairment of the tumor growth factor beta receptor II (TGFBR2) is yet another approach to preventing CAR-T cell exhaustion in the tumor stroma and to gain a long-term effect in pancreatic cancer patient-derived xenografts (PDX). By engineering anti-mesothelin CAR-T cells with CRISPR/Cas9 to knockout the *TGFBR2*, a complete tumor remission was achieved after intratumor or intravenous injection of edited CAR-T cells in the pancreatic tumor PDX mice [54]. Similar results were obtained by PD-1 function impairment in anti-mesothelin CAR-T cells in breast cancer BT-549 orthotopic xenografted mice, where a reduction to complete eradication of xenografted tumors was obtained after intravenous infusion of CRISPR/Cas9 edited CAR-T cells [55]. An augmentation of cytotoxic activity in CAR-T cells was also achieved by CRISPR/Cas9 knockout of diacylglycerol kinase (DGK), which is a negative regulator of CD3 signaling in T cells. Anti-EGFRvIII targeted CAR-T cells electroporated with SpCas9/DGK-gRNA ribonucleoprotein complex showed enhanced cytokine release and cytotoxic effects in human glioblastoma cells line U87 MG in vitro, and in vivo animal models [56]. Another regulator of cytotoxic T cells activity, namely cytotoxic T-lymphocyte associated protein 4 (CTRL4), seems to be also a potential target for enhancing the anti-tumor activity of T cells, as a study shows on primary T cells edited for CTRL4 for treatment, using colorectal cancer HCT-116 as a model [57].

Besides knocking out the PD-1 receptor on T cells to bypass the immune suppressive tumor microenvironment and to circumvent CAR-T cell exhaustion, others focused on targeting instead the programmed death ligand 1 (PD-L1) expressed on the tumor cells by CRISPR/Cas9 to gain a similar tumor inhibition effect. Furthermore, this strategy proved to be efficient to render MDA-MB-231 breast cancer and 786-0 kidney cancer cells susceptible to cisplatin treatment [58], suggesting that the harsh tumor stoma is a major obstacle for both immunotherapies and chemotherapeutic agents. Increased sensitivity to cisplatin treatment was also observed in MDA-MB-231 breast cancer cells by knocking out the PHD finger protein 8 (*PHF8*) gene [59], suggesting that cisplatin resistance has multigenic character, with new genes been validated as potential targets for treatment-refractory cancers. This histone lysine demethylase seems to confer a pan-chemoresistance to malignant cells, as knocking out the *PHF8* gene by CRISPR/Cas9 in the MDA-MB-231 breast cancer tumor cells, not only increased the sensitivity to cisplatin but also polyadenosine diphosphate-ribose polymerase inhibitors (PARPi) both in vitro and in vivo orthotopic tumors [59].

A highly desired goal for cancer theraIy is to achieve a clinical response, with minimal side effects in the non-malignant cells and tissues. The most representative characteristic of cancer cells is the mutation status of diverse genes, both tumor suppressors and oncogenes, making these changes in the primary structure of genes potential targets for therapy. Cleavage of Cas9 nuclease is dependent on PAM recognition and binding [23], and in some instances, in malignant cells mutations can generate PAM sequences by which oncogenes can be specifically targeted. This is the case of the epithelial growth factor receptor (*EGFR*) gene, which is overrepresented in lung cancer cells, with the L878R mutation being distributed in approximately 40% of EGFR-mutated lung cancers [60]. This mutation creates a PAM recognition site for Cas9, making it an appealing target for the specific knockout of L878R-bearing tumors. Researchers have managed to show that L878R mutation can be specifically targeted by CRISPR/Cas9, eventually leading to EGFR expression knockout. A decreased proliferation of human lung cancer H1975 cell line was achieved by targeting L878R mutation, while L878-negative cell lines (i.e., A549) remained unaffected [30,31]. In addition, L878-edited H1978 tumor xenografts exhibited a reduced tumor growth after subcutaneous injection in nude mice [30]. Similarly, intratumor injection of an adenoviral vector that expresses the L878R-gRNA and Cas9 into H1978 and A549 tumor xenografted mice resulted in a decreased tumor growth and prolonged survival rates in H1978 tumor xenografted mice, but not in A549-bearing mice [31], suggesting that such oncogenes mutations can be successfully explored for the development of targeted therapies in lung cancer by CRISPR/Cas9. This targeting approach has been also supported by other studies that take advantage of a mutation in the *KRAS* oncogene, G12S, to specifically inhibit the proliferation of G12S-bearing lung cancer A549 cells [29]. The lung cancer H2228 cell line, which is negative for the G12S mutation, retained its proliferative and tumor growth potential despite being subjected to the same CRISPR/Cas9 treatment as the A549 cell line, both in in vitro and in vivo animal models. The BRAF mutation V600E, which correlates with a poor prognosis in skin melanoma patients [61], has been used for designing a CRISPR/Cas9 system that specifically targets the mutated *BRAF* gene in A375 and G361 melanoma cell lines and mediates cleavage upon stimulation with blue light. The CRISPR/Cas9 design consisted of a blue light dimerization transcriptional activator and a responsive element that drives Cas9 expression. Once the cells are irradiated with blue light, the GAL(65)-VVD-p65 fusion peptide forms an active transcriptional activator that binds the UAS-responsive element, and the Cas9 gene is transcribed. With these innovative controllable CRISPR/Cas9 systems, the authors managed to suppress cell growth, migration, and invasion, and induce apoptosis in the A375 and G361 melanoma cells that harbor the BRAF V600E mutation [62]. Such strategy might also prove to be useful for targeting this mutation in other types of malignant disorders, such as colorectal cancer [63]. Correspondently, others have explored the genomic rearrangements that occur in certain types of cancer, such as Ewing Sarcoma, which give rise to unique fusion oncogenes that can be specifically targeted with CRISPR/Cas9, without side effects and therapy resistance commonly associated with chemotherapeutic agents, such as the tyrosine kinase inhibitor imatinib [64]. Among these, the *EWSR1-FL1* is the most widely spread fusion oncogene in Ewing Sarcoma tumors [65]. Researchers managed to impair *EWSR1-FL1* expression and thus cell proliferation and clonogenic capacity in the A673 cell line in vitro by targeting exon 9 of the *FL1* gene using a gRNA library screening [66]. To reduce the side effects that might result from targeting exonic regions, others focused their research on the intronic regions of the genes involved in the *EWSR1-FL1* expression. By this means, a potent anti-tumor effect, both in vitro and in vivo on A673 tumor xenografted mice models and PDX mice models, has been achieved. Most importantly, deletion of the targeted locus, which is flanked by the two intronic gRNAs occurred only in cells that harbor this particular translocation on the same chromosome. Such deletions were not observed in normal mesenchymal stem cells (MSCs), which are thought to be the cells of origin for human sarcomas [67]. In addition, this targeting approach for oncogenic fusion genes has been successfully used to impair the BCR-ABL1 fusion protein expression in chronic myeloid leukemia K562 cell line, in vitro and on in vivo xenograft athymic nude mice models [67]. With this cell model, others have shown that tumor necrosis factor-alpha (*TNF-α*) gene knockout can markedly reduce the proliferation and clonogenic potential of K562 cells in vitro, and the edited cells displayed impaired tumor xenograft growth in mice models [68]. Furthermore, altered TNF-α function as a result of gene editing was associated with a deregulated metabolism profile. The expression profile of *TNF-α* knockout cells revelated differentially expressed miRNAs involved in the cell cycle, apoptosis, and other pathways associated with the malignant phenotype [68]. Altered metabolism in malignant cells is not something new, and a positive correlation between increased lipid metabolism and cancer development and progression has been established (reviewed in [69,70]). These observations are further supported by the CRISPR/Cas9 knockout study of genes related to fatty acid metabolism. The elongation of very long-chain fatty acid 2 factors (*ELOVL2*) has been pointed to be elevated in renal cell carcinoma (RCC) patients’ tissue, making it a potential target for therapy [71]. CRISPR/Cas9 knockout of *ELOVL2* led to decreased cell proliferation of RCC cell line ACHN and induction of pro-apoptotic related genes in vitro. Even more surprising, ACHN-edited cells failed to produce xenograft tumors in Balb/c mice at the 80–100 days evaluation end period [71]. Genetic screening using a CRISPR gRNA library in colon cancer primary cells highlighted that knocking out three key genes involved in cholesterol biosynthesis (*HMGCR*, *FDPS*, and *GGPS1*) can impair spheroid formation, reduce cancer cell stemness and activate apoptosis. In addition, the edited colon cancer cells also exhibited decreased xenograft tumor growth [72].

The above studies do not cover enti’ely all the efforts researchers have put in to exploit the capabilities of the CRISPR/Cas9 system to knockout oncogenes and potential new therapeutic targets that might translate into a clinical benefit for cancer patients. As listed in Supplementary Table S1, both coding and non-coding genomic sequences as well, such as the ones that encode for miRNAs or long non-coding RNAs (lncRNAs), have been evaluated for CRISPR/Cas9 therapy, due to their pro-carcinogenic role or their propriety to confer resistance to treatment. LncRNAs are particularly hard to knockout, as they lack functional characterization, and complete inactivation of lncRNA genomic locus is necessary to achieve the desired effect. In addition, many lncRNAs are positioned in proximity of coding regions, and collateral, unwanted damage can occur. A recently published protocol describes a simple and effective method to produce genomic knockouts of lncRNAs [73] Trans-acting elements of oncogene expression regulation are among the listed CRISPR/Cas9 targets, such as the case of CCCTC-binding factor (*CTCF*), that creates a loop between the neurotensin (*NTS*) oncogene and an upstream enhancer sequence, promoting an *NTS* overexpression in uveal melanoma tumor cells [74]. It is worth mentioning a particular study [75], in which the authors switched their search for potential CRISPR/Cas9 targets from oncogenes or other sequences with an oncogenic role, to a widespread class of repeat sequences through the human genome, namely the Alu short interspersed nuclear elements (SINE) class of retrotransposons, that number approximately 3 million conserved copies in our genome [76]. By this approach, CRISPR to kill (C2K), the authors aim of generating multiple DSBs throughout the tumor genome, which would make the cancer cell impossible to recover from such an extensive degree of damage. To experimentally validate the C2K system, glioblastoma patient-derived primary cell lines and U87 glioblastoma cell line were transduced with an LV vector expressing the SpCas9 nuclease and a gRNA that targets genomic Alu sequences. The results showed that C2K managed to successfully induce cell-cycle arrest and trigger apoptosis, in addition to synergizing with radiation treatment cell growth inhibition. This system showed to be highly specific for human cells, as no similar effects were obtained on murine cells [75]. However, this study lacks experimental data that would highlight its preclinical application on in vivo glioblastoma models. As both normal and malignant cells alike harbor Alu sequences, tight control of targeted delivery to tumor cells only and restricted malignant cell functionality would be an absolute requirement, to avoid serious side effects.

Despite being a versatile and widely spread technology for gene editing in mammalian cells, using CRISPR/Cas9 comes with some shortcomings, of which undesired “off-target” effects are the most prominent ones [77,78], rendering its clinical applications. As reviewed above, diverse strategies have been employed to overcome this limitation, by exploiting unique features that characterize some malignant disorders. However, targeting a particular feature of one malignant pathology cannot be universally used for other types of cancer. Therefore, diminishing the nuclease function of Cas nuclease is one of the strategies researchers engaged for targeting oncogenes in tumor genomes. Introducing an inactivating mutation, D10A, in the endonuclease RuvC domain of Cas9, renders it as a nickase enzyme (Figure 1B), which cleaves only the non-PAM site due to HNH remaining active domain. By designing two gRNAs that introduce up and downstream nicks cut for each gRNA in each strand, a reduction in “off-target” activity of Cas9 can be achieved [79]. The double nick cuts are repaired similarly to DSB, however, with higher fidelity [80]. This nickase system has been successfully used for achieving an efficient knockdown of miR-146b, which is overexpressed in the anaplastic thyroid cancer (ATC) KTC2 cell line. Transfection of these cells with plasmids encoding for nickase SpCas9 (nSpCas9) and the two gDNAs that target the *MIR146B* genomic locus resulted in reduced proliferation, migration, and clonogenic capacity in vitro. Moreover, with subcutaneous injection of edited KTC2 cells into nude mice, xenografted tumors exhibited a lower growth rate [81]. Correspondently, the plasminogen activator urokinase receptor (*PLAUR*) gene was inactivated by the double-nickase Cas9 system in human melanoma A375p cells, by transfection with nCas9 and two PLAUR-gRNAs plasmids. The obtained results showed a successful impairment of cell growth both in monolayer and in 3D spheroid cultures. Furthermore, edited cells failed to produce xenograft tumors in the NOD SCID mice model, evaluated on day 20 end of the experiment [82].

### 3.2. CRISPR/Cas9 Knockdown of Oncogenes—Transcription Interference Approach

Viewing from a different angle the ability of CRISPR/Cas9 to target a desired genomic locus based on sequence complementarity of the gRNA, additional factors can be recruited to that specific locus (Figure 1E,F). The “off-target” nuclease activity of Cas9, as mentioned above, raises important concerns regarding the safe use of this gene editing technology for clinical applications, therefore researchers depleted Cas9 completely of its nuclease activity, resulting in what is now called dead Cas9 (dCas9, Figure 1G). Using this inactive form of Cas9, and a gRNA that binds the promoter region driving expression of the oncogenic *SNGH3* lncRNA, successful inhibition of cell growth and migration, and induction of apoptosis was achieved in two bladder cancer cell lines, 5637 and SW780 [83]. The mechanism behind the transcriptional repression of *SNGH3* is most probably due to the stearic repulsion of transcription factors that normally would bind the promoter region, which in this case is occupied by the dCas9 protein (Figure 1G). Similarly, others went further for abrogating the expression of oncogenes by fusing the transcriptional repressor Kruppel-associated box (KRAB) and designing tandem gRNAs that bind the coding gene sequence within the first 50–100 bp downstream of the transcription site (Figure 1F). This design limits potential off-target effects, as an unspecific targeting must occur in that narrow 50–100 bp window for efficient transcriptional repression [84]. The study tested the potential therapeutic benefit of such a system, in squamous cell carcinoma of the esophagus and lung, by targeting the ∆Np63 isoform of the *TP63* gene, which is aberrantly expressed in these cancerous tumors and found to be correlated with a clonogenic potential. Transduction of TE8 esophageal squamous cell carcinoma cell line, and lung squamous cell carcinoma EBC2 cell line, with a recombinant adenoviral vector that expresses the fusion protein dCas9-KRAB and a gRNA, an approximately two-fold decrease in the clonogenic potential of the cell line was achieved. Moreover, the TUNEL assay pointed to an elevated degree of apoptosis in the transduced cells [84]. More importantly, ∆Np63 downregulated EBC2 cells were unable to induce tumor xenografts in Balb/c nude mice models. However, a more recent study that targeted the G12S mutation variant of the *KRAS* gene in lung cancer cell line A549 using this CRISPR interference (CRISPRi) system, showed that dCas9-KRAB was outperformed by its wild-type SpCas9 counterpart as a potential therapeutic tool for tumor inhibition [29]. Although a difference in efficiency between Cas9 and dCas9-Krab was observed, a correlation with the previous study that evaluated only dCas9-KRAB [84] is not necessarily relevant, because each study had its own targeting strategy in terms of gRNA design, and also the malignant pathology cell model was different. The therapeutic potential of using dCas9 protein fused to additional functional entities is further supported by other studies, in which epigenetic silencing of the potent *KRAS* oncogene was accomplished by the fusion transcriptional repressor histone deacetylase 1 (HDAC1), which promotes deacetylation of the lysine residues of core histone proteins, leading to a tighter histone–DNA interaction and thus blocking the accessibility of transcription factors to the promoter [85]. This propriety of HDAC1 proved to be successful in knocking down KRAS expression in the colorectal cancer HCT-116 and lung cancer H385 cell line models. This inhibition translated into decreased proliferation and clonogenic potential of the tumor cells, and elevated levels of apoptosis [86]. Such results show the broad spectrum of applications and versatility of CRISPR/Cas9 system, even as non-functional nuclease, for validating potential treatments for malignant disorders.

### 3.3. CRISPR/Cas9 Knock-in of Exogenous DNA

Perhaps one of the far-reaching advantages that the CRISPR/Cas9 system holds over other genome editing methods, is the capability of inserting exogenous DNA cargo to the desired locus, a process mediated by the HDR mechanism of DSB repair (Figure 1C). This process relies on sequence homology between the genomic locus and the donor DNA [23]. Besides CRISPR/Cas9, inserting foreign genetic material can be achieved either by retro- or lentiviral vectors [87,88] or by DNA transposons [89,90], however, the insertion locus is quite random, and even can result in activation of a malignant phenotype, as in the case of lentiviral vectors [91]. Therefore, site-specific targeting and the HDR process represent a powerful tool for achieving directed integration of genes with therapeutic potential for the treatment of malignant disorders. In addition, this strategy proved its usefulness for cancer immunotherapy by disrupting the endogenous T cell receptor (TCR) of primary T cells, with a tumor-reactive engineered TCR. The new *TCR* gene was introduced in the second exon of the endogenous *TRAC* locus by electroporating primary human T cells with Cas9/gRNA RNP, followed by transduction with an AAV vector that carries the engineered *TCR* gene for HDR insertion [92]. Integration into the DSB generated by gRNA and Cas9 abolished TRAC expression, while the new TCR is transcribed by the endogenous promoter. Co-incubation of TCR knocked-in T cells with the HLA-B7 antigen expressing acute myeloid leukemia (CML) ML-2 cells, resulted in tumor cell lysis in vitro, and inhibition of tumor growth in ML-2 tumor xenografts of NSG mice models [92]. Having the possibility of directing insertion of genetic cargo into desired loci, others used the knock-in potential of CRISPR/Cas9 to safely transfer a tumor suppressor gene, the CCCTC-binding factor (*CTCF*) into the AAVS1 integration locus of triple-negative breast cancer cells MDA-MB-231 [93]. As the *CTCF* gene is inactivated in metastatic tumors, its expression in MDA-MB-231 cells led to reduced cell migration in vitro after transfection with double plasmid CRISPR/Cas9-HDR system packed in tumor-targeted nanoparticles. In addition, intravenous injection of edited CTCF knocked-in cells into Balb/c nude mice resulted in the reduced metastatic potential of MDA-MB-231 [93]. Although the AAVS1 site offers a “safe” harbor for the insertion of exogenous DNA, this locus is present in both malignant and normal cells, and the CRISPR/Cas9 alone cannot differentiate between cells on this basis. In this regard, again the break points of tumor-specific genomic rearrangements can be used for targeting malignant cells only. The fusion genes *MAN2A1-FER* found in hepatocellular carcinoma (HCC) and *TMEM135-CCDC67* in prostate cancer (PC) offer the means of designing CRISPR/Cas9 therapies for the insertion of suicidal genes, such as the one for thymidine kinase (*TK*), that catalyzes the conversion of the harmless Ganciclovir to cell toxic compound, therefore mediating tumor cell death [94]. A reduction in cell viability in vitro was observed in edited HCC cell line HUH7, and PC cell lines PC-3 and DU145, exposed to ganciclovir. The tumor cells were previously co-transduced with an adenoviral vector expressing the Cas9 nickase (nCas9) and gRNA targeting the break points of the fusion genes, and a second adenoviral vector for HDR mediated TK insertion. Moreover, these edited cells exhibited impaired xenograft tumor growth in SCID mice after initiation of ganciclovir treatment [94]. Once more, these results highlight the versatility CRISPR/Cas9 technology holds in its simple and humble nature of bacterial origin.

## 4. CRISPR Clinical Trials for the Treatment of Malignant Disorders

With such advancements of technological improvements in CRISPR design meant to overcome each obstacle that might interfere with its clinical translation, until present around 25 clinical trials evaluate the safety and efficiency of using CRISPR/Cas9 system for cancer treatment (Table 1). Among the various strategies for defeating the malignant behavior of cancerous cells in patients, these trials resume phases I or II of clinical testing. The main approaches emphasize the use of autologous transplantation of CRISPR/Cas9 edited immune cells that are engineered to target and impede tumor growth in patients with progressive disease.

In one published phase I clinical trial, the safety and efficiency of CRISPR/Cas9 edited T cells were evaluated on patients with refractory cancer, including two patients with multiple myeloma (MM), and one patient with myxoid/round cell liposarcoma [95] (NCT03399448). In this clinical study, primary T cells from cancer patients were isolated and transfected by electroporation with Cas9 and equimolar quantities of gRNAs targeting the *TRAC* and *TRAB* loci (TCR receptor alpha and beta chains), and the *PDCD1* locus. This was followed by transduction with an LV vector that delivers a synthetic TCR receptor with specificity for a NY-ESO-1 tumor antigen. The engineered T cells were infused back into the donor patient, after lymphodepletion chemotherapy (cyclophosphamide and fludarabine). This multiplex CRISPR/Cas9 targeting impairs the endogenous TCR, thus increasing the tumor reactivity, while the *PDCD1* (PD-1) knockout ensures prolonged persistence in the tumor microenvironment, thus evading the process of T cell exhaustion. The best clinical outcome as a result of this treatment regimen was observed in the sarcoma patient, where an approximately half reduction in the abdominal tumor mass was achieved and persisted for 4 months. One MM patient had stable disease and the other MM progressive disease. Eventually, all patients experienced progressive disease [95]. It is worth noting that no cytokine release syndrome was observed after NY-ESO-1 T cells infusion or other immune-related side effects. Another phase I clinical trial (NCT02793856) evaluated the safety and efficiency of PDCD1 knockout T cells in patients with advanced non-small cell lung cancer (NSCLC). In this study, isolated T cells were collected from NSCLC patients after being subjected to Treg depletion with cyclophosphamide. The PD-1 receptor was knocked out by electroporation with two plasmids, one that encodes the Cas9 nuclease, and the other the gRNA. After selection and expansion, the edited T cells were infused back into the patient bloodstream in escalating three doses. The treatment-related side effects were Grade1/2 and did not include cytokine release syndrome. The median progression-free survival was 7.7 weeks (95% confidence interval, 6.9–8.5 weeks) and the median overall survival was 42.6 weeks (95% confidence interval 10.3–74.9 weeks). The median follow-up was 47.1 weeks (13.4 to 116.0 weeks) [96].

These published results show that CRISPR/Cas9 technology can be safely used to address malignant disorders; however, more advanced and efficient editing platforms are needed to achieve a better clinical outcome.

## 5. Concluding Remarks and Future Perspective

Trying to capture the existing data of CRISPR/Cas9-based therapeutic approaches for malignant diseases, both in pre-clinical and clinical setups, we have encountered a vast array of studies, that can be grouped into three main categories: studies that aim of knocking out oncogenes, studies that use an impaired Cas9 nuclease fused with other functional entities to silence oncogenes, and studies that use the HDR mechanism repair of DSBs generated by Cas9 nuclease to insert a genetic cargo (Supplementary Table S1). However, most of them fall in the first category, suggesting that such an approach is more reliable for developing treatment strategies for cancer. Moreover, knocking out genes by small insertions and deletions (indels) were among the pioneer studies that used CRISPR/Cas9 in mammalian cells [23]. Therefore, the scientific community holds a wider experience of using the NHEJ mechanism for inducing frameshifts in the coding sequence of genes, and hence impairment of function. New CRISPR/Cas9 designs emerged as a consequence of the small “imperfections” this technology comes with, namely the off-target effects. The human genome is a wide “genetic field” for this new engineering tool, and collateral damage can occur when this technology is applied for a specific goal. Therefore, researchers invested a great deal of effort for narrowing down these unwanted side effects by different means. Firstly, they partially ablated the nuclease activity to achieve a higher specificity; however, this requires additional gRNAs to have both DNA stranded nicked. Further, researchers envisioned a dead nuclease Cas9 that no longer can be used for its primary function, but rather as a tool to prevent transcription of the target genes by blocking the transcription factors to bind the genes’ promoter, or to prevent elongation of the transcribed genes. To augment the inhibition effect of dead nuclease Cas9, different peptides have been fused that act in a similar manner, namely, to block the binding sites of transcription factors and prevent gene expression.

An elegant approach to accomplish a tumor-specific gene inactivation explored the unique genomic rearrangements that occur in the tumor genome, resulting in distinct gene fusions. Therefore, the breakpoint of such fusions can be used to design gRNAs that target the fusion point of the gene, and this strategy proved to be efficient for specific gene knockout in tumor cells that harbor such genetic rearrangements [66,67]. In addition, some point mutations of oncogenes can lead to a new PAM motif sequence, which can be used for oncogene targeting with a gRNA that shares sequence homology next to that PAM sequence. Equally important, the fusion breakpoints that characterizes some malignant cells has been used by researchers to insert exogenous genetic cargo, such as suicide genes, that will eventually be activated upon HDR-mediated insertion between the breakpoint of the fusion genes [94]. Though, one must consider that fusion oncogenes are highly heterogenic within a cohort of patients, as some studies suggest. For example, the *MAN2A1-FER* fusion oncogene was found to be present in 15.7% of a cohort of hepatocellular carcinoma patients [97], while a second study points out that 78.8% of the analyzed clinical samples were positive for this fusion oncogene [98]. For the *TMTM135-CCDC67* fusion, the investigated samples cohort consisted of 11 fusion positive out of 213, meaning less than 1% of all samples [99]. Therefore, a pre-screening for the fusion genes would be necessary to identify which patients could benefit from such a treatment approach. Furthermore, the knock-in potential of CRISPR/Cas9 requires more advanced delivery systems for carrying the genetic editing machinery, because, in addition to the basic Cas9 nuclease and gRNAs, other genetic elements are needed for the HDR mechanism of exogenous DNA insertion into the genome. Therefore, no story is too good to be true, as each new accomplishment comes with additional efforts to be invested for making that story written history. What history does tell us, is that the malignant cell is the most evolved cell that managed to escape death by any means possible, and indeed, this ability is given by its constant plasticity to adapt to new challenges. At the molecular level, the malignant cell is characterized by increased genomic instability, and when combined with its high proliferation rates, new, more evolved, and adapted cells can arise, and lead eventually to tumor relapse. This is a lesson that every clinician knows too well and must deal with in everyday practice with cancer patients.

As described above, many studies explore CRISPR/Cas9 technology as a potential treatment for cancer, and few studies did reach clinical setups, and by an ex vivo approach, meaning cells are retrieved from the patient’s body, engineered with CRISPR/Cas9, and reintroduced back into the patient. This tells us that our enthusiasm for making CRISPR/Cas9 therapy a reality remains at the level of knowledge and observation. Perhaps reaching our vision of genome engineering for cancer treatment could mean a wider range of genes should be targeted to accomplish a full therapeutic effect. Further, some studies mentioned in this paper support the concept of CRISPR multiplexing as an attainable therapeutic approach [72,100,101]. It is worth mentioning a particular study, where the authors used CRISPR/Cas9 was used not for knocking out a gene, or two, three genes, but a highly repetitive sequence, the Alu short interspersed nuclear element (SINE), that numbers more than a million copies spread throughout the human genome. Although this simple, yet powerful CRISPR/Cas9 system was tested only on glioblastoma cells in vitro with positive results [75], it introduces a new concept of “dirty CRISPR/Cas9”, that aims to cut the tumor genome to such an extent that it cannot recover from such extensive damage. In addition, further combination with DNA damaging agents can have a devastating effect on the tumor cell. However, a careful design of delivery strategies is mandatory to avoid deleterious side effects, as Alu SINE retrotransposons are also conserved in normal cells. In this regard, an innovating platform for co-delivery CRISPR/Cas9 and small molecule drugs has been developed based on mesoporous silica nanoparticles that are loaded with the small molecule inhibitor axitinib, locked with CRISPR/Cas9 RNP complex targeting the PD-L1 receptor, and further encapsulated in a PEGylated lipid shell. Biodistribution of these nanoparticles shows preferential accumulations in tumors, where the reducing intracellular environment triggers the release of the CRISPR/Cas9 and subsequently of the axitinib. An enhanced reduction in mouse melanoma xenograft tumor growth was achieved, and prolonged survival rates were also observed in mice treated with these nanoparticles [102].

Finally, the preclinical tumor models used for evaluating the efficiency of CRISPR/Cas9 for cancer treatment are of utmost importance to successfully translate this technology into clinical use. As listed in Supplementary Table S1, few in vivo studies have a prolonged follow-up end time of the treatment outcome, which can offer important information on whether a tumor relapse will occur despite the initial treatment response. Such an outcome is not something new for clinicians, as many cancer patients experience tumor remission after treatment, and later tumor relapse and progressive disease. Therefore, the step from laboratory testing to the clinical use of CRISPR-based therapeutic is still a major one for the scientific community and great efforts are still needed to pursue such a goal. An equally important player for moving CRISPR/Cas9 technology from “bench to bedside” is the delivery system for the component elements of the genome editing machinery, which must be stable in plasma for systemic administration and highly specific for targeting tumor cells, while sparing the healthy ones, and thus minimizing side effects. Moreover, the delivery vector must evade an immune clearance to reach the targeted cells. Despite the numerous non-viral and viral-based strategies developed for CRISPR/Cas9 delivery, few have reached clinical trials and mostly rely on ex vivo editing of immune cells and autologous/allogenic transplantation for enhancing anti-tumor proprieties of these cells in the patient’s body. This story of delivery systems for CRISPR/Cas9 has been expanded as a book chapter by Chira and colleagues [103]. 

## Figures and Tables

**Table 1:** List of clinical trials using CRISPR technology for cancer treatment.

Pathological Condition	Phase	Status	CRISPR-Engineered Therapeutic Agent	Other Therapies	Identifier
B-cell acute lymphoblastic leukemia	Phase 1	Recruiting	Allogenic transplantation of engineered T cells—PBLTT52CAR19	-	NCT04557436
CD19^+^ leukemia and lymphoma	Phase 1	Withdrawn	Allogenic transplantation of engineered T cells—PACE CART19	-	NCT05037669
Gastrointestinal cancers	Ph½ 1/2	Recruiting	Autologous transplantation of CISH CRISPR TILs	Chemotherapy—cyclophosphamide and fludarabine Immunotherapy—aldesleukin	NCT04426669
HIV-infected subjects with hematological malignances	N/A	Unknown	Allogenic transplantation of CRISPR/Cas9 CCR5 gene modified CD34+ hematopoietic stem/progenitor cells	-	NCT03164135
Human papillomavirus-related malignant neoplasm	Phase 1	Unknown	Local direct application of HPV16 E6/E7T1 or CRISPR/Cas9-HPV18 E6/E7T2	-	NCT03057912
Epstein-Barr virus (EBV)-associated malignancies	Ph½ 1/2	Recruiting	Autologous transplantation of PD-1 knockout EBV-CTL cells	Chemotherapy—cyclophosphamide and fludarabine Immunotherapy—interleukin-2	NCT03044743
Non-small cell lung cancer	Phase 1	Completed	Autologous transplantation of PD-1 knockout T cells	Chemotherapy—cyclophosphamide	NCT02793856
Renal cell carcinoma	Phase 1	Withdrawn (no funding)	Autologous transplantation of PD-1 knockout T cells	Chemotherapy—cyclophosphamide Immunotherapy—interleukin-2	NCT02867332
Prostate cancer	Phase 1	Withdrawn (no funding or financial support	Autologous transplantation of PD-1 knockout T cells	Chemotherapy—cyclophosphamide Immunotherapy—interleukin-2	NCT02867345
Bladder cancer	Phase 1	Withdrawn (no funding)	Autologous transplantation of PD-1 knockout T cells	Chemotherapy—cyclophosphamide Immunotherapy—interleukin-2	NCT02863913
Hepatocellular carcinoma	Phase 1	Recruiting	Autologous transplantation of PD-1 knockout T cell	TACE	NCT04417764
Esophageal cancer	Phase 1	Completed	Autologous transplantation of PD-1 knockout T cells	-	NCT03081715
CD19^+^ leukemia and lymphoma	Ph½ 1/2	Recruiting	Allogenic transplantation of UCART019	-	NCT03166878
Leukemia and lymphoma	Ph½ 1/2	Recruiting	Allogenic transplantation of CAR-T Cells Targeting CD19 and CD20 or CD22	-	NCT03398967
T or B-cell malignancies	Phase 1	Recruiting	Allogenic transplantation of CTX130	Prior lymphodepleting chemotherapy	NCT04502446
B-cell malignancies	Phase 1	Recruiting	Allogenic transplantation of CTX110	Prior lymphodepleting chemotherapy	NCT04035434
Solid tumors	Phase 1	Unknown	Mesothelin-directed CAR-T cells	Prior conditioning regimen of paclitaxel and cyclophosphamide	NCT03747965
Renal cell carcinoma	Phase 1	Recruiting	Allogenic transplantation of CTX130	Prior lymphodepleting chemotherapy	NCT04438083
Multiple myeloma	Phase 1	Recruiting	Allogenic transplantation of CTX120	Prior lymphodepleting chemotherapy	NCT04244656
Solid tumors	Phase 1	Recruiting	Anti-mesothelin CAR-T cells	-	NCT03545815
Multiple myeloma Melanoma Synovial sarcoma Myxoid/round cell liposarcoma	Phase 1	Terminated	Autologous T cells were transduced with a lentiviral vector to express NY-ESO-1 and electroporated with CRISPR guide RNA to disrupt the expression of endogenous TCRα, TCRβ, and PD-1 (NYCE T Cells)	Cyclophosphamide Fludarabine	NCT03399448
Relapsed or refractory CD19^+^ leukemia or lymphoma	Phase 1	Recruiting	Autologous T cells engineered to specify CD19 transduced with a lentiviral vector and electroporated with CRISPR guide RNA to disrupt the expression of endogenous HPK1 administered by IV injection	Cyclophosphamide Fludarabine	NCT04037566
Non-Hodgkin lymphoma	Phase 1	Recruiting	CRISPR-edited allogeneic CAR-T cell therapy targeting CD19	Cyclophosphamide Fludarabine	NCT04637763
Acute myeloid leukemia	Ph½ 1/2	Recruiting	Autologous WT1-directed TCR T cells engineered ex vivo using CRISPR/Cas9	Pre-conditioning chemotherapy: cyclophosphamide Fludarabine	NCT05066165

## Data Availability

Not applicable.

## Supplementary Materials

The following supporting information can be downloaded at: https://www.mdpi.com/article/10.3390/cells11182781/s1, Table S1: CRISPR/Cas gene targets for the treatment of malignant disorders, and the experimental setup (in vivo/ex vivo and in vivo) used for gene editing (knockout, knockdown, knock-in) with the outcome of the experiment. References [104,105,106,107,108,109,110,111,112,113,114,115,116,117,118,119,120,121,122,123,124,125] are cited in Supplementary Materials File.
