# Supplementary material for: Genome Editing Approaches with CRISPR/Cas9 for Cancer Treatment: Critical Appraisal of Preclinical and Clinical Utility, Challenges, and Future Research
Source: Cells. 2022 Sep 6;11(18):2781. doi: 10.3390/cells11182781 (PMC9496708; doi:10.3390/cells11182781)
Supplement: Supplementary file 1 [file cells-11-02781-s001.zip › cells-1837304-supplementary.pdf]

Supplementary

Table S1. CRISPR gene targets for treatment of malignant disorders, and the experimental setup (*in vivo/ex vivo* and *in vivo*) used to gene editing (knockout, knockdown, knock-in) with the outcome of the experiment

CRISPR/ Cas system	Target genes (Gene editing)	Function	*In vitro/ex vivo*		*In vivo*		Ref.
			Experimental setup	Outcome	Experimental setup	Outcome	
Acute Myeloid Leukemia							
SpCas9	*TCR* ^{KI}	cell receptor	Electroporation of *ex vivo* primary T cells with Cas9/gRNA-TRAC RNP and subsequent transduction with AAV6-TCR-HDR viral vector<br>Co-incubation of TCR knock-in T cells with ML-2 AML cells	Cell lysis of ML-2 cells expressing HLA-B7 antigen	Intravenous injection of edited T cells into ML-2 tumor xenografted NSG mice	ML-2 tumor xenograft rejection	[1]
Bladder cancer							
Cas9	*PVT1* ^{KO}<br>*ANRIL* ^{KO}<br>*(CDKN2B-AS1)*	oncogene lncRNA	Co-transfection of T24 and 5637 cells with DOX-inducible Cas9 and gRNA-PVT1 or gRNA-ANRIL expressing plasmids	Induction of apoptosis, reduced proliferation, and migration	N/A	N/A	[2]
Cas13a	*SMAD7e* ^{KD}	oncogene	Transfection of T24 and 5637 cells with Cas13a/gRNA expressing plasmid	Inhibition of estrogen dependent tumor cells proliferation and migration; induction of apoptosis	Subcutaneous injection of edited T24 cells into Balb/c nude mice	Reduced growth of T24 tumor xenografts at day 30 end point	[3]
Cas13d	*MYC* ^{KD}	oncogene	Transfection of T24 and 5637 cells with hTERT-sensing engineered Cas13d/gRNA expressing plasmid	Suppression of proliferation, migration, and invasion; induction of apoptosis	N/A	N/A	[4]

dCas9	*SNGH3* ^{KD}	lncRNA	Transfection of 5637 and SW780 cells with dCas9/gRNA RNP	Suppression of cell growth and invasion; elevated apoptosis	N/A	N/A	[5]
Cas9	*HNRNPU* ^{KD}	drug resistance	Genome-wide lentiCRISPR-gRNA library screening in T24 cells treated with cisplatin	Cell cycle arrest in S-phase; apoptosis; induction of cisplatin sensitivity; increase of apoptosis	Subcutaneous injection of T24 edited cells into Balb/c nu/nu mice treated with cisplatin	No significant increase in tumor mass at day 28 end point	[6]
Breast cancer							
SpCas9	*PD-1* ^{KO} *(PDCD1)*	cell receptor	Electroporation of *ex vivo* mesothelin-targeted CAR-T cells with Cas9/gRNA RNP, and co-culture with BT-549 cells	Increased tumor cell lysis; elevated IFNγ and IL-2; reduced CAR-T cells exhaustion	Intravenous injection of PD-1 mesothelin-targeted CAR-T cells into BT-549 tumor orthotopic xenografted NSG mice	Reduction to eradication of established tumor xenografts at day 44 after treatment	[7]
Cas9	*PHF8* ^{KO}	oncogene	Co-transfection of MDA-MB-231 cells with Cas9 and gRNA expressing plasmids	Increased sensitivity to PARPi and cisplatin	Orthotopic injection of edited MDA-MB-231 cells into mammary fat pad of SCID mice	Decreased tumor growth at day 17 end point after PARPi initiation treatment	[8]
Cas9	*CTCF* ^{KI}	tumor-suppressor	Transfection of MDA-MB-231 cells with dual plasmid Cas9/gRNA-AAVS1 and CTCF-HDR system packed in targeted polymeric nanoparticles	Inhibition of cell migration	Intravenous injection of edited MDA-MB-231 cells into Balb/c nude mice	Reduced metastatic potential	[9]
SpCas9	*CD274* ^{KO} *(PD-L1/ B7-H1)*	oncogene	Transfection of MDA-MB-231 cells with Cas9/gRNA expressing plasmid	Increased sensitivity to cisplatin, induction of apoptosis	Subcutaneous injection of edited MDA-MB-231 cells into NOD SCID mice	Reduction of tumor growth; increased sensitivity to cisplatin	[10]
SpCas9	*RILP* ^{KO}	oncogene	Co-transduction of MDA-MB-231 and MCF-7 cells with LV-DOX-inducible Cas9 and LV-gRNA-RILP expressing viral vectors	Inhibition of cell survival, proliferation and tumorigenicity upon DOX induction	Subcutaneous injection of edited MDA-MB-231 cells into athymic nude mice	Inhibition of tumor growth and angiogenesis	[11]

Cervical cancer							
Cas9	*HPV18-E6* ^{KO}	oncogene	Transduction of Cas9-expressing HeLa, HCS-2, and SKG-I cells with AAV-gRNA viral vector	Induction of apoptosis, reduced proliferation	Intratumor injection of AAV-gRNA into Cas9-expressing SKG-I-xenografted Balb/c nude mice	Reduction in tumor mass measured at day 42 end point after treatment	[12]
SpCas9	*HPV16-E6* ^{KO}	oncogene	Transduction of SiHa and Caski cells with HCAdV-Cas9/gRNA viral vector	Activation of p53 and induction of apoptosis, decreased cell proliferation	N/A	N/A	[13]
SaCas9	*HPV18-E6* ^{KO}	oncogene	Transduction of HeLa cells with AVV/Cas9/gRNA viral vector	Increased cell apoptosis, reduced cell proliferation	N/A	N/A	[14]
Cas9	*MLL5* ^{KO} *(KMT2E)*<br>*HPV18-E6* ^{KO}	oncogenes	Transfection of HeLa cells with Cas9/gRNA-MLL5 or -E6 expressing plasmid	Reduced cell viability, increased sensibility to Cisplatin	N/A	N/A	[15]
SpCas9 ^{KO}	*TERT* ^{KO} *(mutant: exon 4 deletion)*	oncogene	Electroporation of HeLa cells with Cas9/gRNA expressing plasmid	Reduced cell growth and increase cell death	Intramuscular injection of $TERT^{+/-}$ edited Hela cells into Nude mice	Impairment of $TERT^{+/-}$ Hela xenograft tumor growth	[16]
SpCas9	*HPV18-E6* ^{KO}<br>*HPV18 E7* ^{KO}	oncogenes	Transfection of HeLa cells with an autocatalytic Cas9/gRNA-E6 or -E7 plasmid system	Inhibition of cell proliferation and motility	N/A	N/A	[17]
SpCas9	*HPV16-E6* ^{KO}<br>*HPV16-E7* ^{KO}<br>*HPV18-E6* ^{KO}<br>*HPV18-E7* ^{KO}	oncogenes	Co-transfection of HeLa and Caski cells with Cas9 and gRNA expressing plasmids	Reduction of cell viability, activation of p53 pathway	Intravenous injection of Cas9 and gRNA-16E7 or 18E7 expression plasmids packed in PEGylated liposomes at multiple time points	Eradication of Caski xenograft tumors in Rag1 mice (gRNA-16E7) at day 77 after treatment<br>Inhibition of HeLa xenograft tumors in Rag1 mice (gRNA-18E7) at day 46 after treatment	[18]

Cas9	*HPV16-E6/HPV16-E7/PD-1* ^{TKO} *(PDCD1)*	oncogene, cell receptor	Co-transfection of SiHa cells with Cas9 and gRNA-E6/E7/PD-1expressing plasmids	Increased apoptosis and reduced viability in E6/E7 knockout SiHa cells; decreased expression of PD-L1	In situ electroporation of Cas9 and gRNAs (E6/E7/PD1) plasmids into SiHa orthotopic uterus tumors established in human PMBC inoculated SCID mice (hu-PBL-SCID)	Inhibition of tumor growth evaluated at day 20 after E6/E7/PD1 knockout treatment; increased survival at day 80-100 end point	[19]
			Chondrosarcoma				
SpCas9 ^{KO}	*SOX9* ^{KO}	oncogene	Transfection of SW1353 cells with Cas9/gRNA expressing plasmid	Reduced proliferation, clonogenicity and migration; increased adhesion, apoptosis and polyploidy; increased sensitivity to doxorubicin	N/A	N/A	[20]
			Chronic myeloid leukemia				
SpCas9	*BCR-ABL1* ^{KO} *(ABL1)*	fusion oncogene	Electroporation of K562 cells with LV-Cas9/gRNA expressing plasmid	Decrease of clonogenic potential; increase of apoptosis	Intratumor injection of Ad-Cas9/gRNA into K562 tumor xenografted athymic nude mice	Reduction of tumor mass at day 30 end point after cell inoculation and vector treatment; increased survival at day 64 end point follow-up	[21]
SpCas9	*TNF* ^{KO} *(TNF-α)*	oncogene	Electroporation of K562 cells with Cas9/gRNA expressing plasmid	Impaired proliferation and clonogenic capacity; increased sensitivity to imatinib	Subcutaneous injection of edited K562 cells into Balb/c mice pretreated with cyclophosphamide	Reduction of xenograft tumor growth at day 21 after subcutaneous cell injection	[22]
			Colorectal cancer				
dCas9-HDAC1	*KRAS* ^{KD}	oncogene	Co-transfection of HCT-116 cells with dCas9-HDAC1 expressing plasmid and gRNA-KRAS; or dCas9-HDAC1/gRNA-KRAS RNP	Inhibition of proliferation, clonogenic capacity; increased cell death	N/A	N/A	[23]

SpCas9	*CASP3* ^{KO}	oncogene	Transduction of HCT-116 cells with LV-Cas9/gRNA expressing viral vector	Reduced cell growth and proliferation; increased sensitivity to mitomycin C and radiation.	Intravenous injection of edited HCT-116 cells into nude mice<br>Subcutaneous injection of edited HCT-116 cells into nude mice	Reduced metastatic potential in intravenous injected mice at day 32 end point<br>Reduced invasiveness and metastatic potential in subcutaneous injected mice	[24]
SpCas9	*CTLA4* ^{KO}	cell receptor	Transduction of primary cytotoxic T cells LV-Cas9/gRNA expressing viral vector	Decreased HCT-116 cell viability; elevated apoptosis rate; increased cytokines release	Intravenous injection of edited cytotoxic T cells into HCT-116 tumor xenografted SCID mice	Decreased tumor growth at week 7 end point	[25]
SpCas9	*HMGCR/ FDPS* ^{DKO}	cholesterol synthesis	LentiCRISPR-gRNA library screening in patient derived cancer stem cell spheroids	Impairment of sphere formation; reduced cell stemness; increase of apoptosis	Subcutaneous injection of edited cancer stem cells spheroids into nude mice	Inhibition of xenograft tumor growth at day 20-25 end point	[26]
Cas9	*KRAS* ^{KO}	oncogene	Transfection of SW-480 cells with Cas9/gRNA RNP polymeric nanoparticles	Inhibition of cell proliferation and induction of apoptosis	Intravenous injection of Cas9/gRNA RNP polymeric nanoparticles into SW-480 tumor xenografted Balb/c mice	Inhibition of xenograft tumor growth at day 21 end point after treatment; reduction of metastatic potential at day 14 endpoint after treatment	[27]
Endometrial cancer							
Cas9	*LIFR* ^{KO}	oncogene	Transfection of Cas9-expressing Ishikawa and AN3-CA cells with gRNA	Reduced cell viability and clonogenic potential	Subcutaneous injection of edited Ishikawa cells into SCID mice	Inhibition of tumor growth at day 30-40 end point	[28]
Esophageal cancer							
dCas9-KRAB	*TP63* ^{KD} *(mutant*: $\Delta Np63$ *isoform)*	oncogene	Transduction of TE8 and KYSE70 cells with Ad-dCas9-KRAB/gRNA expressing viral vector	Reduction of clonogenic capacity	N/A	N/A	[29]
Glioblastoma							

SpCas9	*DGKB* ^{KO} *(DGK)*	DAG metabol.	Electroporation of ex vivo EGFRvIII targeted CAR-T cells with Cas9/gRNA RNP and co-culture with EGFRvIII-positive U87-MG cells	Increased anti-tumor toxicity and cytokine release	Subcutaneous injection of U87-MG cells into NSG mice and infusion of edited CAR-T cells at day 28 and 30 post-inoculation, followed by adjuvant TMZ treatment	Increased tumor regression at day 56 end point; increased number of tumor-infiltrating T cells	[30]
SpCas9	*Alu SINE* ^{DSB}	retro-transposon	Transduction of U87 cells and glioblastoma patient-derived primary cells with LV-Cas9/gRNA expressing viral vector	Suppression of cell growth; cell cycle G2/M arrest; induction of apoptosis; increased sensitivity to apoptosis	N/A	N/A	[31]
Cas9	*PDCD1* ^{KO} *(PD1/ PD-1)*	T cell receptor	Electroporation of human PBMC with Cas9/gRNA expressing plasmid and subsequent transduction with anti-EGFRvIII CAR-T LV expressing viral vector; co-culture with EGFRvIII-expressing DK-MG cells	Inhibition of cell proliferation	N/A	N/A	[32]
Kidney cancer							
SpCas9	*CD274* ^{KO} *(PD-L1/ B7-H1)*	oncogene	Transfection of 786-0 cells with Cas9/gRNA expressing plasmid	Increased sensitivity to cisplatin, induction of apoptosis	N/A	N/A	[10]
Liver cancer							
Cas13a	*TERT/EZH2/ RelA* $^{IKD/TKD}$	oncogenes	Transfection of HepG2 cells with Cas13a/gRNA-TERT/EZH/RelA expressing plasmids<br>Transduction of HepG2 cells with AAV-Cas13a/gRNA-TERT/EZH/RelA expressing viral vector	Increased apoptosis at higher level by triple knockdown compared with individual knockdown	N/A	N/A	[33]

Cas9	*PD-1* ^{KO} *(PDCD1)*	cell receptor	Electroporation of *ex vivo* GPC3 targeted CAR T cells with Cas9/gRNA RNP and co-culture with GPC3-expressing PLC/PRF/5 cells	Enhanced cytokine release and cytotoxic effects	Intravenous injection of edited GPC3-CAR T cells into PLC/PRF/5 tumor xenografted NSG mice	Inhibition of tumor growth; increase survival at day 20 after treatment	[34]
SpCas9-D10A	*MAN2A1 -FER* ^{KI}	fusion oncogene	Co-transduction of HUH7 cells with Ad-Cas9-D10A/gRNA expressing viral vector and donor Ad-TK-HDR, followed by Ganciclovir treatment	Increased cell death	Subcutaneous injection of edited HUH7 into SCID mice, followed by intraperitoneal Ganciclovir injection	Reduction of tumor mass at week 8 end point after Ganciclovir treatment	[35]
Lung cancer							
dCas9-HDAC1	*KRAS* ^{KD}	oncogene	Co-transfection of H358 cells with dCas9-HDAC1 expressing plasmid and gRNA-KRAS; or dCas9-HDAC1/gRNA-KRAS RNP	Inhibition of proliferation, clonogenic capacity; increased cell death	N/A	N/A	[23]
Cas9	*MAPK7* ^{KO} *(ERK5)* *MAP2K5* ^{KO} *(MEK5)*	oncogene	Co-transfection of H1975 and A549 cells with Cas9 and gRNA-ERK5 or -MEK5 expressing plasmids	Reduced proliferation rate	Injection of ERK5 or MEK5 edited H460 cells into Balb/c mice	Reduced tumor growth at day 16-18 end point	[36]
SpCas9	*EGFR* ^{KO} *(mutant: L858R)*	oncogene	Co-transduction of H1975 cells with Ad-Cas9 and Ad-gRNA expressing viral vectors	Reduced cell proliferation and viability	Intratumor injection of Cas9 and gRNA-EGFRmut Ad vectors into H1975 or A549 tumor xenografted nude mice	Inhibition of tumor growth and increased survival rate at day 31 end point	[37]
SpCas9	*EGFR* ^{KO} *(mutant: L858R)*	oncogene	Transduction of H1975 cells with LV-Cas9/gRNA expressing viral vector	Decrease of cell proliferation and clonogenic capacity	Subcutaneous injection of edited H1975 cells into Balb/c nude mice	Reduction of tumor burden	[38]
LbCas12a	*EGFR* ^{KO}	oncogene	Transduction of A549 or H1299 cells with oncolytic Ad-Cas9/gRNA expressing viral vector	Reduced cell viability	Intratumor injection of oncolytic Ad/Cas9/gRNA-EGFR expressing viral vector into A549 tumor xenografted nude mice	Inhibition of tumor growth at day 35 end point	[39]

SpCas9<br>dCas9-KRAB	*KRAS* $^{KO, KD}$ *(mutant: G12S)*	oncogene	Transduction of A549 cells with LV-Cas9/gRNA expressing vector; transduction of A549 cells with dCas9-KRAB/gRNA-G12S LV expressing vector	Inhibition of cell proliferation; cell cycle arrest in S phase; reduced clonogenic capacity	Intratumor injection of Ad/Cas9/gRNA vector or LV/dCas9-KRAB/gRNA-G12S into A549 tumor xenografted nude mice	Inhibition of tumor weight at day 28 end point	[40]
Cas9	*TGFBR2* ^{KO}	oncogene	Electroporation of *ex vivo* mesothelin targeted CAR-T cells with Cas9/gRNA, and co-culture with CRL5826 cells	Increased cell lysis in the absence of TGFβ	Intratumor or intravenous injection of edited CAR-T cells into CRL5828 tumor xenografted NPG mice	Complete tumor eradication after intratumor injection at day 49 end point; inhibition of tumor growth after intravenous at day 73 end point	[41]
dCas9-KRAB	*TP63* ^{KD} *(mutant*: $\Delta Np63$ *isoform)*	oncogene	Transduction of EBC2 cells with Ad-dCas9-KRAB/gRNA-p63 expressing viral vector	Reduction of clonogenic capacity; increased apoptosis	Subcutaneous injection of edited EBC2 cells into BALB/c nude mice	Suppression of xenografted tumor growth	[29]
Cas13a	*EML4-ALK* ^{KD}	fusion oncogene	Co-transfection of H3122 and H2228 cells with Cas13a and gRNA-EML4-ALK expressing plasmids	Reduced cell viability	N/A	N/A	[42]
Melanoma (uveal)							
Cas9	CTCF-binding site ^{DEL}	NTS oncogene enhancer	Co-transfection of OCM1 cells with Cas9 and gRNA expression plasmids	Downregulation of MAPK, Rap1 and PI3K-Akt pathways; reduced clonogenic capacity	Subcutaneous injection of edited OCM1 cells into nude mice; subarachnoid injection of LV-edited OCM1 cells.<br>Intravenous injection of LV-edited OCM1 cells	Reduced tumor growth in subcutaneous xenografted mice at day 28 end point; Reduced tumor weight in orthotopic xenografted mice; prolonged survival in orthotopic xenografted mice; reduced number of metastatic sites in intravenous injected mice	[43]
Melanoma (skin)							

Cas9	*BRAF* ^{KO} *(mutant: V600E)*	oncogene	Transfection of A375 and G361 cells with a blue light-inducible Cas9/gRNA expressing plasmid	Reduced cell proliferation, cell migration and invasion, and increased apoptosis upon blue light stimulation	N/A	N/A	[44]
Cas9	*PTGS2* ^{KO} *(COX-2)*	oncogene	Co-transfection of A2058 cells with Cas9/gRNA expressing plasmid and reporter HDR-insertion plasmid	Reduced cell migration and invasion; impaired clonogenic and spheroid formation capacity	Subcutaneous injection of edited A2085 cells into NMRI-nude mice	Reduced tumor growth	[45]
nCas9 (D10A nickase)	*PLAUR* ^{KO}	oncogene	Transfection of A375p cells with nCas9/gRNA expression plasmid	Impaired cell growth in 2D and spheroid 3D cultures	Subcutaneous injection of edited A375p cells into NOD SCID mice	Marked tumor growth inhibition at day 20 end point	[46]
Cas9	*SNCA* ^{KO}	oncogene	Transfection of SK-Mel-28 cells with Cas9/gRNA expressing plasmid	Inhibition of cell proliferation; decrease of TfR1 expression; increase of ferritin and ROS	Subcutaneous injection of edited Sk-Mel-28 cells into NOD SCID mice	Decreased tumor xenograft growth at day 72 end point; decreased TfR1 and FPN1 expression, and increase of ferritin and DMT1 in excised tumor tissue	[47]
Cas9	*SIRT6* ^{KO}	oncogene	Selection of KO clones from a commercial A375 SIRT6 KO cell pool	Impaired cell growth and clonogenic potential; decreased viability; altered expression of angiogenesis, apoptosis and EMT related genes	Subcutaneous injection of edited A375 cells into athymic mice	Inhibition of tumor growth evaluated at day 26 end point	[48]
Multiple myeloma							
SpCas9	*CDK9* ^{KO}	oncogene	Transduction of OPM2 cells with LV-Cas9/gRNA expressing viral vector	Inhibition of cell growth; reduced expression levels of p42/p55, Mcl1, and increased expression levels of c-PARP and Casp3	N/A	N/A	[49]
Oral cancer							

Cas9	*BSG* ^{KO} *(CD147)*	oncogene	Transfection of Cal27 cells with Cas9/gRNA expression plasmid, and subsequent doxorubicin treatment	Decreased cell proliferation, migration, and invasiveness; increased sensitivity to doxorubicin; decreased expression levels of angiogenesis and metastasis-related genes; increase of apoptosis-related genes	Subcutaneous injection of edited Cal27 cells into nude mice, followed by doxorubicin treatment	Increased sensitivity to doxorubicin and inhibition of tumor growth in BSG-KO and doxorubicin	[50]
Osteosarcoma							
SpCas9	*TP53* ^{KO} *(mutant)*	oncogene	Transfection of KHOS and KHOSR2 cells with Cas9/gRNA expressing plasmid, and subsequent doxorubicin treatment	Reduced cell proliferation, migration, and invasion; decreased expression level of Bcl-2, Survivin, and IGF-1R; increased sensitivity to doxorubicin	N/A	N/A	[51]
Ovarian cancer							
Cas9	*PLK1* ^{KO}	oncogene	N/A	N/A	Intraperitoneal injection of Cas9/gRNA RNP encapsulated anti-EGRF lipid nanoparticle into Hsd: Athymic Nude-Foxn1nu mice, pre-inoculated intraperitoneal with OV8 tumor cells	Inhibition of tumor growth and increased survival rates at day 43 end point after treatment	[52]
SpCas9	*MTF1* ^{KO}	oncogene	Transduction of SKOV3 and OVCAR3 cells with LV-Cas9/gRNA expressing vector	Reduced cell proliferation, migration, and invasiveness; upregulation of R-cadherin and KRT7; downregulation of SNAI2 and B-catenin;	N/A	N/A	[53]
Pancreatic cancer							

Cas9	*HIF1A* ^{KO} *(HIF-1α)*	oncogene	Transfection of BxPC-3 cells with R8-dGR targeted nanolipoplexes loaded with Cas9/gRNA expressing plasmid and w/o paclitaxel	Decreased proliferation in HIF-1 KO cells; enhanced sensitivity to paclitaxel	Intravenous injection of R8-dGR targeted nanolipoplexes loaded with Cas9/gRNA expressing plasmid and w/o paclitaxel, into subcutaneous BxPC-3 tumor xenografted mice;<br>Intravenous injection of R8-dGR targeted nanolipoplexes loaded with Cas9/gRNA expressing plasmid and w/o paclitaxel into BxPC-3 metastatic mice (intravenous inoculation)	Inhibition of xenograft tumor growth at day 7 end point after treatment (HIF-1 KO and paclitaxel); increased survival rates in metastatic models at day 30-40 end point	[54]
CasRx	*KRAS* ^{KD} *(mutant: G12D)*	oncogene	Transduction of PANC-1 and AsPC-1 cells with LV-CasRx/gRNA expressing viral vector, and subsequent treatment of with gemcitabine	Inhibition of proliferation, cell growth and clonogenic potential in PANC-1 cells; increased sensitivity to gemcitabine	For xenograft tumor model: subcutaneous injection of edited PANC-1 cells into Balb/c mice;<br>For orthotopic model: injection of AsPC-1 cells into pancreas of Balb/c mice; subsequent intraperitoneal injection of AAV8/CasRx/gRNA expressing viral vector w/o further gemcitabine treatment;<br>For PDX model: subcutaneous implantation of patient tumor tissue fragments into non-obese diabetic SCID mice; subsequent intraperitoneal injection of AAV8/CasRx/gRNA expressing viral vector w/o	Inhibition of tumor growth and increased sensitivity to gemcitabine in xenograft model; increased survival rate in orthotopic model gemcitabine treated at day 100 end point; inhibition of PDX tumor growth w/o gemcitabine treatment	[55]

					further gemcitabine treatment		
Cas9	*TGFBR2* [KO]	oncogene	N/A	N/A	Electroporation of *ex vivo* mesothelin targeted CAR-T cells with Cas9/gRNA RNP, and subsequent intratumor or intravenous injection of edited CAR-T cells into PDX NPG mice	Complete PDX tumor eradication after intratumor or intravenous injection at day 42 end point after treatment	[41]
Prostate cancer							
SpCas9	*FOXA1* [KO]	oncogene	Transfection of LNCaP cells with Cas9/gRNA expressing plasmid	Increased expression of pro-apoptotic genes, cell cycle progression and epithelial phenotype associated genes	N/A	N/A	[56]
nSpCas9-D10A	*TMEM135-DEUP1* [KI] *(CCDC67)*	fusion oncogene	Co-transduction of PC-3 and DU145 cells with Ad-nCas9/gRNA expressing viral vector and donor Ad-TK-HDR, followed by Ganciclovir treatment	Increased cell death	Subcutaneous injection of edited PC-3 and DU145 cells into SCID mice, followed by intraperitoneal Ganciclovir injection	Reduction of tumor mass at week 4 end point after treatment; inhibition of metastasis; increased survival at day 30 after treatment	[35]
Renal cancer (kidney)							
SpCas9	*ELOVL2* [KO]	lipid metabol.	Transfection of ACHN cells with Cas9/gRNA expressing plasmid	Decreased cell proliferation; increased expression levels of pro-apoptotic genes a decreased level of anti-apoptotic genes	Subcutaneous injection of edited ACHN cells into Balb/c mice	Impairment or complete suppression of tumor xenograft at day 80-100 end point	[57]
Sarcoma (Ewings)							
SpCas9	*EWSR1-FLI1* [KO]	fusion oncogene	Transduction of A673 and RD-ES cells with LV-Cas9/gRNA expressing viral vector	Inhibition of cell proliferation, survival and conogenicity	Intratumor injection of Ad-Cas9/gRNA expressing viral vector into A673 xenografted tumors of athymic mice;	Inhibition of A673 tumor xenograft growth at day 25 end point after treatment; prolonged survival in A673 xenografted mice at day	[21]

					Intratumor injection of Ad -Cas9/gRNA expressing vector into PDX tumors of athymic mice	80; inhibition of PDX tumor growth at day 28 end point after treatment; prolonged survival at day 70 end point	
SpCas9	*EWSR1-FLI1* ^{KO}	fusion oncogene	Transduction of A653 cells with LV-Cas9 expressing viral vector, followed by transduction with LV-RNA expressing viral vector	Inhibition of cell proliferation; induction of senescence	N/A	N/A	[58]
Thyroid cancer (squamous cell carcinoma)							
SpCas9	*HDAC1/ HDAC2* $^{IKO/DKO}$	oncogenes	Transduction of SW579 cells with LV-Cas9 and LV-gRNA-HDAC1+/-HDAC2 expressing viral vectors	Increased cell death only in double knockout transduced cells	N/A	N/A	[59]
Thyroid cancer (anaplastic carcinoma)							
nSpCas9-D10A	*miR-146B* ^{KD}	oncogenic microRNA	Transfection of KTC1 cells with nCas9/gRNA expressing plasmid	Reduction of cell proliferation, migration and clonogenicity	Subcutaneous injection of edited KTC1 cells into nude mice	Inhibition of tumor growth	[60]

Abbreviation for gene editing mechanisms:

DEL – deletion
DKO – double knockout
DSB – double-strand break
IKD – individual knockdown
IKO – individual knockout
KD – knockdown
KI – knock-in
KO – knockout
TKD – triple knockdown
TK – thymidine kinase

Abbreviation for gene names:

ABL1 (BCR-ABL1) – ABL Proto-Oncogene 1, Non-Receptor Tyrosine Kinase
ALK – ALK Receptor Tyrosine Kinase
Alu SINE – Alu Short interspersed nuclear elements
BRAF – B-Raf Proto-Oncogene, Serine/Threonine Kinase
BSG (CD147) – Basigin (Ok Blood Group)
CASP3 – Caspase 3
CD274 (PD-L1/ B7-H1) – CD274 Molecule, Programmed Death Ligand 1
KRAS – Kirsten Rat Sarcoma Proto-Oncogene
LIFR – Leukemia Inhibitory Factor Receptor Subunit Alpha
MAN2A1 – Mannosidase Alpha Class 2A Member 1
MAP2K5 (MEK5) – Mitogen-Activated Protein Kinase Kinase 5
MAPK7 (ERK5) – Mitogen-Activated Protein Kinase 7
MIR146B – MicroRNA 146b
MTF1 – Metal Regulatory Transcription Factor 1
MYC – MYC Proto-Oncogene, BHLH Transcription Factor

CDK9 – Cyclin Dependent Kinase 9
CDKN2B-AS1 (ANRIL) - CDKN2B Antisense RNA 1
CTCF – CCCTC-Binding Factor
CTLA4 – Cytotoxic T-Lymphocyte Associated Protein 4
DEUP1 (CCDC67) – Deuterosome Assembly Protein 1
DGKB (DGK) – Diacylglycerol Kinase Beta
EGFR – Epidermal Growth Factor Receptor
ELOVL2 – ELOVL Fatty Acid Elongase 2
EML4 – EMAP Like 4
EWSR1 – EWS RNA Binding Protein 1
EZH2 – Enhancer of Zeste 2 Polycomb Repressive Complex 2 Subunit
FDPS – Farnesyl Diphosphate Synthase
FER – FER Tyrosine Kinase
FLI1 – Fli-1 Proto-Oncogene, ETS Transcription Factor
FOXA1 – Forkhead Box A1
HDAC1 – Histone Deacetylase 1
HDAC2 – Histone Deacetylase 2
HIF1A (HIF-1α) – Hypoxia Inducible Factor 1 Subunit Alpha
HMGCR – 3-Hydroxy-3-Methylglutaryl-CoA Reductase
HNRNPU – Heterogeneous Nuclear Ribonucleoprotein U
HPV16 E6 / HPV18 E6 – Human Papilloma Virus 16/18 serotype E6-associated protein
HPV16 E7 / HPV18 E7 – Human Papilloma Virus 16/18 serotype E7-associated protein
KMT2E (MLL5) – Lysine Methyltransferase 2E (Inactive)
KRAS – KRAS Proto-Oncogene, GTPase
PDCD1 (PD1/ PD-1) – Programmed Cell Death 1
PHF8 – PHD Finger Protein 8
PLAUR – Plasminogen Activator, Urokinase Receptor
PLK1 – Polo Like Kinase 1
PTGS2 (COX-2) – Prostaglandin-Endoperoxide Synthase 2
PVT1 – Plasmacytoma Variant Translocation 1
RELA (RelA) – RELA Proto-Oncogene, NF-KB Subunit
RILP – Rab Interacting Lysosomal Protein
SIRT6 – Sirtuin 6
SMAD7e – SMAD Family Member 7 enhancer RNA
SNCA – Synuclein Alpha
SNGH3 – Small Nucleolar RNA Host Gene 3
SOX9 – SRY-Box Transcription Factor 9
TCR – T Cell Receptor
TERT – Telomerase Reverse Transcriptase
TGFBR2 – Transforming Growth Factor Beta Receptor 2
TMEM135 – Transmembrane Protein 135
TNF (TNF-α) – Tumor Necrosis Factor
TP53 – Tumor Protein P53
TP63 – Tumor Protein P63

Other abbreviations:

AAV – adenoassociated virus
AAVS1 – AAV integration site 1
AML – acute myeloid leukemia
dCas9 – dead Cas9
GPC3- glypican 3
HCAdV – high-capacity adenovirus
HDR – homology directed recombination
LV – lentivirus
metabol. – metabolism
nCas9 – nickase Cas9
PARPi – poly (ADP-ribose) polymerase inhibitor
RNP – ribonucleoprotein
TMZ – temozolomide
w/o – with or without

References

1. Albers, J.J.; Ammon, T.; Gosmann, D.; Audehm, S.; Thoene, S.; Winter, C.; Secci, R.; Wolf, A.; Stelzl, A.; Steiger, K.; et al. Gene Editing Enables T -Cell Engineering to Redirect Antigen Specificity for Potent Tumor Rejection. *Life Science Alliance* **2019**, *2*, e201900367, doi:10.26508/lsa.201900367.

2. Peng, L.; Pan, P.; Chen, J.; Yu, X.; Wu, J.; Chen, Y. A Tetracycline-inducible CRISPR / Cas9 System, Targeting Two Long Non-coding RNAs , Suppresses the Malignant Behavior of Bladder Cancer Cells. *Oncology Letters* **2018**, doi:10.3892/ol.2018.9157.

3. Che, W.; Ye, S.; Cai, A.; Cui, X.; Sun, Y. CRISPR - Cas13a Targeting the Enhancer RNA - SMAD7e Inhibits Bladder Cancer Development Both in Vitro and in Vivo. *Frontiers in Molecular Biosciences* **2020**, *7*, 607740, doi:10.3389/fmolb.2020.607740.

4. Zhuang, C.; Zhuang, C.; Zhou, Q.; Huang, X.; Gui, Y.; Lai, Y.; Yang, S. Engineered CRISPR / Cas13d Sensing hTERT Selectively Inhibits the Progression of Bladder Cancer In Vitro . *Frontiers in Molecular Biosciences* **2021**, *8*, 646412, doi:10.3389/fmolb.2021.646412.

5. Cao, Y.; Hu, Q.; Zhang, R.; Li, L.; Guo, M.; Wei, H.; Zhang, L.; Wang, J.; Li, C. Knockdown of Long Non -Coding RNA SNGH3 by CRISPR - dCas9 Inhibits the Progression of Bladder Cancer . *Frontiers in Molecular Biosciences* **2021**, *8*, 657145, doi:10.3389/fmolb.2021.657145.

6. Shi, Z.; Hao, L.; Han, X.; Wu, Z.-X.; Pang, K.; Dong, Y.; Qin, J.; Wang, G.; Zhang, X.; Xia, T.; et al. Targeting HNRNPU to Overcome Cisplatin Resistance in Bladder Cancer. *Molecular Cancer* **2022**, *21*, 37, doi:10.1186/s12943-022-01517-9.

7. Hu, W.; Zi, Z.; Jin, Y.; Li, G.; Shao, K.; Cai, Q.; Ma, X.; Wei, F. CRISPR / Cas9 -Mediated PD -1 Disruption Enhances Human Mesothelin-Targeted CAR T Cell Effector Functions. *Cancer Immunology, Immunotherapy* **2019**, *68*, 365–377, doi:10.1007/s00262-018-2281-2.

8. Ma, S.; Zhang, J.; Guo, Q.; Cao, C.; Bao, K.; Liu, L.; Chen, C.D.; Liu, Z.; Yang, J.; Yang, N.; et al. Disrupting PHF8 - TOPBP1 Connection Elicits a Breast Tumor-Specific Vulnerability to Chemotherapeutics. *Cancer Letters* **2022**, *530*, 29–44, doi:10.1016/j.canlet.2022.01.010.

9. Duan, J.; Bao, C.; Xie, Y.; Guo, H.; Liu, Y.; Li, J.; Liu, R.; Li, P.; Bai, J.; Yan, Y.; et al. Targeted Core-Shell Nanoparticles for Precise CTCF Gene Insert in Treatment of Metastatic Breast Cancer. *Bioactive Materials* **2022**, *11*, 1–14, doi:10.1016/j.bioactmat.2021.10.007.

10. Wu, X.; Li, Y.; Liu, X.; Chen, C.; Harrington, S.M.; Cao, S.; Xie, T.; Pham, T.; Mansfield, A.S.; Yan, Y.; et al. Targeting B7 - H1 (PD - L1) Sensitizes Cancer Cells to Chemotherapy. *Heliyon* **2018**, *4*, e01039, doi:10.1016/j.heliyon.2018.e01039.

11. Singhal, J.; Chikara, S.; Horne, D.; Awasthi, S.; Salgia, R.; Singhal, S.S. Targeting RLIP with CRISPR / Cas9 Controls Tumor Growth. *Carcinogenesis* **2021**, *42*, 48–57, doi:10.1093/carcin/bgaa048.

12. Yoshiba, T.; Saga, Y.; Urabe, M.; Uchibor, R.; Matsubara, S.; Fujiwara, H.; Mizukami, H. CRISPR / Cas9 -mediated Cervical Cancer Treatment Targeting Human Papillomavirus E6 . *Oncology Letters* **2018**, doi:10.3892/ol.2018.9815.

13. Ehrke-Schulz, E.; Heinemann, S.; Schulte, L.; Schiwon, M.; Ehrhardt, A. Adenoviral Vectors Armed with PAPILLOMAVIRUs Oncogene Specific CRISPR / Cas9 Kill Human - Papillomavirus - Induced Cervical Cancer Cells . *Cancers* **2020**, *12*, 1934, doi:10.3390/cancers12071934.

14. Noroozi, Z.; Shamsara, M.; Valipour, E.; Esfandyari, S.; Ehghaghi, A.; Monfaredan, A.; Azizi, Z.; Motevaseli, E.; Modarressi, M.H. Antiproliferative Effects of AAV -Delivered CRISPR / Cas9 -Based Degradation of the HPV18 - E6 Gene in HeLa Cells. *Scientific Reports* **2022**, *12*, 2224, doi:10.1038/s41598-022-06025-w.

15. Pirouzfar, M.; Amiri, F.; Dianatpour, M.; Takhshid, M.A. CRISPR / Cas9 -Mediated Knockout of MLL5 Enhances Apoptotic Effect of Cisplatin in HeLa Cells in Vitro. *EXCLI journal* **2020**, *19*, 170–182, doi:10.17179/excli2019-1957.

16. Wen, L.; Zhao, C.; Song, J.; Ma, L.; Ruan, J.; Xia, X.; Chen, Y.E.; Zhang, J.; Ma, P.X.; Xu, J. CRISPR / Cas9 - Mediated TERT Disruption in Cancer Cells . *International Journal of Molecular Sciences* **2020**, *21*, 653, doi:10.3390/ijms21020653.

17. Tian, R.; Liu, J.; Fan, W.; Li, R.; Cui, Z.; Jin, Z.; Huang, Z.; Xie, H.; Li, L.; Huang, Z.; et al. Gene Knock-out Chain Reaction Enables High Disruption Efficiency of HPV18 E6 / E7 Genes in Cervical Cancer Cells. *Molecular Therapy - Oncolytics* **2022**, *24*, 171–179, doi:10.1016/j.omto.2021.12.011.

18. Jubair, L.; Fallaha, S.; McMillan, N.A.J. Systemic Delivery of CRISPR / Cas9 Targeting HPV Oncogenes Is Effective at Eliminating Established Tumors . *Molecular Therapy* **2019**, *27*, 2091–2099, doi:10.1016/j.ymthe.2019.08.012.

19. Zhen, S.; Lu, J.; Liu, Y.-H.; Chen, W.; Li, X. Synergistic Antitumor Effect on Cervical Cancer by Rational Combination of PD1 Blockade and CRISPR - Cas9 -Mediated HPV Knockout. *Cancer Gene Therapy* **2020**, *27*, 168–178, doi:10.1038/s41417-019-0131-9.

20. Stöckl, S.; Lindner, G.; Li, S.; Schuster, P.; Haferkamp, S.; Wagner, F.; Prodinger, P.M.; Multhoff, G.; Boxberg, M.; Hillmann, A.; et al. SOX9 Knockout Induces Polyploidy and Changes Sensitivity to Tumor Treatment Strategies in a Chondrosarcoma Cell Line . *International Journal of Molecular Sciences* **2020**, *21*, 7627, doi:10.3390/ijms21207627.

21. Martinez-Lage, M.; Torres-Ruiz, R.; Puig-Serra, P.; Moreno-Gaona, P.; Martin, M.C.; Moya, F.J.; Quintana-Bustamante, O.;

Garcia-Silva, S.; Carcaboso, A.M.; Petazzi, P.; et al. In Vivo CRISPR / Cas9 Targeting of Fusion Oncogenes for Selective Elimination of Cancer Cells. *Nature Communications* **2020**, *11*, 5060, doi:10.1038/s41467-020-18875-x.

22. Shen, N.; Liu, S.; Cui, J.; Li, Q.; You, Y.; Zhong, Z.; Cheng, F.; Guo, A.-Y.; Zou, P.; Yuan, G.; et al. Tumor Necrosis Factor \α Knockout Impaired Tumorigenesis in Chronic Myeloid Leukemia Cells Partly by Metabolism Modification and miRNA Regulation. *OncoTargets and Therapy* **2019**, *Volume 12*, 2355–2364, doi:10.2147/OTT.S197535.

23. Liu, J.; Sun, M.; Cho, K.B.; Gao, X.; Guo, B. A CRISPR - Cas9 Repressor for Epigenetic Silencing of KRAS . *Pharmacological Research* **2021**, *164*, 105304, doi:10.1016/j.phrs.2020.105304.

24. Zhou, M.; Liu, X.; Li, Z.; Huang, Q.; Li, F.; Li, C.-Y. Caspase-3 Regulates the Migration, Invasion and Metastasis of Colon Cancer Cells: Metastasis of Colon Cancer Cells. *International Journal of Cancer* **2018**, *143*, 921–930, doi:10.1002/ijc.31374.

25. Shi, L.; Meng, T.; Zhao, Z.; Han, J.; Zhang, W.; Gao, F.; Cai, J. CRISPR Knock out CTLA -4 Enhances the Anti-Tumor Activity of Cytotoxic T Lymphocytes. *Gene* **2017**, *636*, 36–41, doi:10.1016/j.gene.2017.09.010.

26. Gao, S.; Soares, F.; Wang, S.; Wong, C.C.; Chen, H.; Yang, Z.; Liu, W.; Go, M.Y.Y.; Ahmed, M.; Zeng, Y.; et al. CRISPR Screens Identify Cholesterol Biosynthesis as a Therapeutic Target on Stemness and Drug Resistance of Colon Cancer. *Oncogene* **2021**, *40*, 6601–6613, doi:10.1038/s41388-021-01882-7.

27. Wan, T.; Chen, Y.; Pan, Q.; Xu, X.; Kang, Y.; Gao, X.; Huang, F.; Wu, C.; Ping, Y. Genome Editing of Mutant KRAS through Supramolecular Polymer-Mediated Delivery of Cas9 Ribonucleoprotein for Colorectal Cancer Therapy. *Journal of Controlled Release* **2020**, *322*, 236–247, doi:10.1016/j.jconrel.2020.03.015.

28. Tang, W.; Ramasamy, K.; Pillai, S.M.A.; Santhamma, B.; Konda, S.; Pitta Venkata, P.; Blankenship, L.; Liu, J.; Liu, Z.; Altwegg, K.A.; et al. LIF / LIFR Oncogenic Signaling Is a Novel Therapeutic Target in Endometrial Cancer. *Cell Death Discovery* **2021**, *7*, 216, doi:10.1038/s41420-021-00603-z.

29. Yoshida, M.; Yokota, E.; Sakuma, T.; Yamatsuji, T.; Takigawa, N.; Ushijima, T.; Yamamoto, T.; Fukazawa, T.; Naomoto, Y. Development of an Integrated CRISPRi Targeting Δ Np63 for Treatment of Squamous Cell Carcinoma. *Oncotarget* **2018**, *9*, 29220–29232, doi:10.18632/oncotarget.25678.

30. Jung, I.-Y.; Kim, Y.-Y.; Yu, H.-S.; Lee, M.; Kim, S.; Lee, J. CRISPR / Cas9 - Mediated Knockout of DGK Improves Antitumor Activities of Human T Cells . *Cancer Research* **2018**, *78*, 4692–4703, doi:10.1158/0008-5472.CAN-18-0030.

31. Głów, D.; Maire, C.L.; Schwarze, L.I.; Lamszus, K.; Fehse, B. CRISPR -to- Kill (C2K)– Employing the Bacterial Immune System to Kill Cancer Cells . *Cancers* **2021**, *13*, 6306, doi:10.3390/cancers13246306.

32. Nakazawa, T.; Natsume, A.; Nishimura, F.; Morimoto, T.; Matsuda, R.; Nakamura, M.; Yamada, S.; Nakagawa, I.; Motoyama, Y.;

Park, Y.-S.; et al. Effect of CRISPR / Cas9 - Mediated PD -1- Disrupted Primary Human Third - Generation CAR - T Cells Targeting EGFRvIII on In Vitro Human Glioblastoma Cell Growth . *Cells* **2020**, *9*, 998, doi:10.3390/cells9040998.

33. Gao, J.; Luo, T.; Lin, N.; Zhang, S.; Wang, J. A New Tool for CRISPR - Cas13a - Based Cancer Gene Therapy . *Molecular Therapy - Oncolytics* **2020**, *19*, 79–92, doi:10.1016/j.omto.2020.09.004.

34. Guo, X.; Jiang, H.; Shi, B.; Zhou, M.; Zhang, H.; Shi, Z.; Du, G.; Luo, H.; Wu, X.; Wang, Y.; et al. Disruption of PD -1 Enhanced the Anti -Tumor Activity of Chimeric Antigen Receptor T Cells Against Hepatocellular Carcinoma . *Frontiers in Pharmacology* **2018**, *9*, 1118, doi:10.3389/fphar.2018.01118.

35. Chen, Z.-H.; Yu, Y.P.; Zuo, Z.-H.; Nelson, J.B.; Michalopoulos, G.K.; Monga, S.; Liu, S.; Tseng, G.; Luo, J.-H. Targeting Genomic Rearrangements in Tumor Cells through Cas9 -Mediated Insertion of a Suicide Gene. *Nature Biotechnology* **2017**, *35*, 543–550, doi:10.1038/nbt.3843.

36. Sánchez-Fdez, A.; Re-Louhau, M.F.; Rodríguez-Núñez, P.; Ludeña, D.; Matilla-Almazán, S.; Pandiella, A.; Esparís-Ogando, A. Clinical, Genetic and Pharmacological Data Support Targeting the MEK5 / ERK5 Module in Lung Cancer. *npj Precision Oncology* **2021**, *5*, 78, doi:10.1038/s41698-021-00218-8.

37. Koo, T.; Yoon, A.-R.; Cho, H.-Y.; Bae, S.; Yun, C.-O.; Kim, J.-S. Selective Disruption of an Oncogenic Mutant Allele by CRISPR / Cas9 Induces Efficient Tumor Regression. *Nucleic Acids Research* **2017**, *45*, 7897–7908, doi:10.1093/nar/gkx490.

38. Cheung, A.H.-K.; Chow, C.; Zhang, J.; Zhou, Y.; Huang, T.; Ng, K.C.-K.; Or, T.C.-T.; Yao, Y.Y.; Dong, Y.; Fung, J.M.-W.; et al. Specific Targeting of Point Mutations in EGFR L858R -Positive Lung Cancer by CRISPR / Cas9 . *Laboratory Investigation* **2018**, *98*, 968–976, doi:10.1038/s41374-018-0056-1.

39. Yoon, A.-R.; Jung, B.-K.; Choi, E.; Chung, E.; Hong, J.; Kim, J.-S.; Koo, T.; Yun, C.-O. CRISPR - Cas12a with an oAd Induces Precise and Cancer - Specific Genomic Reprogramming of EGFR and Efficient Tumor Regression . *Molecular Therapy* **2020**, *28*, 2286–2296, doi:10.1016/j.ymthe.2020.07.003.

40. Gao, Q.; Ouyang, W.; Kang, B.; Han, X.; Xiong, Y.; Ding, R.; Li, Y.; Wang, F.; Huang, L.; Chen, L.; et al. Selective Targeting of the Oncogenic \textit KRAS G12S Mutant Allele by CRISPR / Cas9 Induces Efficient Tumor Regression. *Theranostics* **2020**, *10*, 5137–5153, doi:10.7150/thno.42325.

41. Tang, N.; Cheng, C.; Zhang, X.; Qiao, M.; Li, N.; Mu, W.; Wei, X.-F.; Han, W.; Wang, H. TGF -β Inhibition via CRISPR Promotes the Long-Term Efficacy of CAR T Cells against Solid Tumors. *JCI Insight* **2020**, *5*, e133977, doi:10.1172/jci.insight.133977.

42. Saifullah; Sakari, M.; Suzuki, T.; Yano, S.; Tsukahara, T. Effective RNA Knockdown Using CRISPR - Cas13a and

Molecular Targeting of the EML4 - ALK Transcript in H3122 Lung Cancer Cells . *International Journal of Molecular Sciences* **2020**, *21*, 8904, doi:10.3390/ijms21238904.

43. Chai, P.; Yu, J.; Jia, R.; Wen, X.; Ding, T.; Zhang, X.; Ni, H.; Jia, R.; Ge, S.; Zhang, H.; et al. Generation of Onco-Enhancer Enhances Chromosomal Remodeling and Accelerates Tumorigenesis. *Nucleic Acids Research* **2020**, *48*, 12135–12150, doi:10.1093/nar/gkaa1051.

44. Wu, X.; Huang, H.; Yu, B.; Zhang, J. A Blue Light - Inducible CRISPR - Cas9 System for Inhibiting Progression of Melanoma Cells . *Frontiers in Molecular Biosciences* **2020**, *7*, 606593, doi:10.3389/fmolb.2020.606593.

45. Haase-Kohn, C.; Laube, M.; Donat, C.K.; Belter, B.; Pietzsch, J. CRISPR / Cas9 Mediated Knockout of Cyclooxygenase -2 Gene Inhibits Invasiveness in A2058 Melanoma Cells . *Cells* **2022**, *11*, 749, doi:10.3390/cells11040749.

46. Biagioni, A.; Chillà, A.; Del Rosso, M.; Fibbi, G.; Scavone, F.; Andreucci, E.; Peppicelli, S.; Bianchini, F.; Calorini, L.; Li Santi, A.; et al. CRISPR / Cas9 uPAR Gene Knockout Results in Tumor Growth Inhibition , EGFR Downregulation and Induction of Stemness Markers in Melanoma and Colon Carcinoma Cell Lines . *Frontiers in Oncology* **2021**, *11*, 663225, doi:10.3389/fonc.2021.663225.

47. Shekoohi, S.; Rajasekaran, S.; Patel, D.; Yang, S.; Liu, W.; Huang, S.; Yu, X.; Witt, S.N. Knocking out Alpha-Synuclein in Melanoma Cells Dysregulates Cellular Iron Metabolism and Suppresses Tumor Growth. *Scientific Reports* **2021**, *11*, 5267, doi:10.1038/s41598-021-84443-y.

48. Garcia-Peterson, L.M.; Ndiaye, M.A.; Chhabra, G.; Singh, C.K.; Guzmán-Pérez, G.; Iczkowski, K.A.; Ahmad, N. CRISPR/Cas9-Mediated Knockout of SIRT6 Imparts Remarkable Anti-Proliferative Response in Human Melanoma Cells in Vitro and in Vivo. *Photochemistry and photobiology* **2020**, *96*, 1314, doi:10.1111/PHP.13305.

49. Zhang, Y.; Zhou, L.; Leng, Y.; Dai, Y.; Orlowski, R.Z.; Grant, S. Positive Transcription Elongation Factor b (P - TEFb) Is a Therapeutic Target in Human Multiple Myeloma. *Oncotarget* **2017**, *8*, 59476–59491, doi:10.18632/oncotarget.19761.

50. Pan, S.; Su, Y.; Sun, B.; Hao, R.; Gao, X.; Han, B. Knockout of CD147 Inhibits the Proliferation, Invasion, and Drug Resistance of Human Oral Cancer CAL27 Cells in Vitro and in Vivo . *International Journal of Biological Macromolecules* **2021**, *181*, 378–389, doi:10.1016/j.ijbiomac.2021.03.102.

51. Tang, F.; Min, L.; Seebacher, N.A.; Li, X.; Zhou, Y.; Hornicek, F.J.; Wei, Y.; Tu, C.; Duan, Z. Targeting Mutant TP53 as a Potential Therapeutic Strategy for the Treatment of Osteosarcoma. *Journal of Orthopaedic Research* **2019**, *37*, 789–798, doi:10.1002/jor.24227.

52. Rosenblum, D.; Gutkin, A.; Kedmi, R.; Ramishetti, S.; Veiga, N.; Jacobi, A.M.; Schubert, M.S.; Friedmann-Morvinski, D.; Cohen,

Z.R.; Behlke, M.A.; et al. CRISPR - Cas9 Genome Editing Using Targeted Lipid Nanoparticles for Cancer Therapy. *Science Advances* **2020**, *6*, eabc9450, doi:10.1126/sciadv.abc9450.

53. Ji, L.; Zhao, G.; Zhang, P.; Huo, W.; Dong, P.; Watari, H.; Jia, L.; Pfeffer, L.M.; Yue, J.; Zheng, J. Knockout of MTF1 Inhibits the Epithelial to Mesenchymal Transition in Ovarian Cancer Cells . *Journal of Cancer* **2018**, *9*, 4578–4585, doi:10.7150/jca.28040.

54. Li, M.; Xie, H.; Liu, Y.; Xia, C.; Cun, X.; Long, Y.; Chen, X.; Deng, M.; Guo, R.; Zhang, Z.; et al. Knockdown of Hypoxia-Inducible Factor-1 Alpha by Tumor Targeted Delivery of CRISPR / Cas9 System Suppressed the Metastasis of Pancreatic Cancer. *Journal of Controlled Release* **2019**, *304*, 204–215, doi:10.1016/j.jconrel.2019.05.019.

55. Jiang, W.; Li, H.; Liu, X.; Zhang, J.; zhang, W.; Li, T.; Liu, L.; Yu, X. Precise and Efficient Silencing of Mutant Kras $^ \textrm G12D $ by CRISPR - CasRx Controls Pancreatic Cancer Progression. *Theranostics* **2020**, *10*, 11507–11519, doi:10.7150/thno.46642.

56. Albayrak, G.; Konac, E.; Ugras Dikmen, A.; Bilen, C.Y. FOXA1 Knock-out via CRISPR / Cas9 Altered Casp -9, Bax , CCND1 , CDK4 , and Fibronectin Expressions in LNCaP Cells. *Experimental Biology and Medicine* **2018**, *243*, 990–994, doi:10.1177/1535370218791797.

57. Tanaka, K.; Kandori, S.; Sakka, S.; Nitta, S.; Tanuma, K.; Shiga, M.; Nagumo, Y.; Negoro, H.; Kojima, T.; Mathis, B.; et al. ELOVL2 Promotes Cancer Progression by Inhibiting Cell Apoptosis in Renal Cell Carcinoma. *Oncology Reports* **2021**, *47*, 23, doi:10.3892/or.2021.8234.

58. Cervera, S.T.; Rodríguez-Martín, C.; Fernández-Tabanera, E.; de Mera, R.M.; Morin, M.; Fernández-Peñalver, S.; Iranzo-Martínez, M.; Amhih-Cardenas, J.; García-García, L.; González-González, L.; et al. Therapeutic Potential of EWSR1 – FLI1 Inactivation by CRISPR / Cas9 in Ewing Sarcoma . *Cancers* **2021**, *13*, 3783, doi:10.3390/cancers13153783.

59. Lin, C.-L.; Tsai, M.-L.; Lin, C.-Y.; Hsu, K.-W.; Hsieh, W.-S.; Chi, W.-M.; Huang, L.-C.; Lee, C.-H. HDAC1 and HDAC2 Double Knockout Triggers Cell Apoptosis in Advanced Thyroid Cancer . *International Journal of Molecular Sciences* **2019**, *20*, 454, doi:10.3390/ijms20020454.

60. Santa-Inez, D.C. de; Fuziwara, C.S.; Saito, K.C.; Kimura, E.T. Targeting the Highly Expressed microRNA miR -146b with CRISPR / Cas9n Gene Editing System in Thyroid Cancer . *International Journal of Molecular Sciences* **2021**, *22*, 7992, doi:10.3390/ijms22157992.
